# misosoup: a metabolic modeling tool for identifying minimal microbial communities, facilitates the exploration of microbial ecology and biotechnological applications

**DOI:** 10.1128/msystems.00688-26

**Published:** 2026-07-28

**Authors:** Nicolas Ochsner, Magdalena San Román, Adrián Jiménez-Fernández, Sebastian Bonhoeffer, Alberto Pascual-Garcia

**Affiliations:** 1 Department of Environmental Systems Sciences, ETH 197815, Zürich, Switzerland; 2 Centro Nacional de Biotecnología, CSIC 54447, Madrid, Spain; 3 Basque Culinary Center, Faculty of Gastronomy Sciences, Mondragon Unibersitatea 385941, Donostia–San Sebastián, Spain; Boston College, Chestnut Hill, Massachusetts, USA; Karlsruher Institut fur Technologie, Karlsruhe, Germany

**Keywords:** microbial consortia, metabolic cross-feeding, microbial biotechnology, microbial ecology, genome-scale metabolic modeling, constraint-based modeling, niche expansion, functional groups, synthetic ecology, microbial cooperation

## Abstract

Microbial survival and function often depend on metabolic interactions within communities. Therefore, a central question in disentangling microbial organization is determining which minimal groups of strains are able to thrive in a given medium—referred to as “minimal communities.” Answering this question is essential for understanding microbial distribution, enhancing laboratory cultivation, and designing synthetic communities (SynComs). Here, we introduce misosoup, a Python package for identifying minimal communities (minimal supplying community search). Through genome-scale constraint-based metabolic modeling, misosoup enables the systematic identification of communities that support microbial growth in environments where individual strains fail to survive alone. We validate misosoup against experimentally verified minimal communities, demonstrating its ability to predict known cooperative interactions, cocultures, and consortia with biotechnological potential. We further illustrate the use of misosoup to investigate broad microbial ecology questions by applying it to a set of 60 marine microbes, finding pervasive cross-feeding-driven niche expansion, and showing how the detailed outputs provided by misosoup facilitate research on hot topics such as the identification of functional groups. In summary, misosoup provides a powerful tool for microbial ecology and community design, with potential applications in both research and biotechnological innovation.

**IMPORTANCE:**

Microbes often rely on each other to survive, especially in environments where they cannot live alone. Understanding which small groups of microbes can thrive together—called minimal communities—is key to improving laboratory research, designing synthetic ecosystems, and exploring how microbes spread in nature. To support this, we developed misosoup, a Python tool that identifies these communities using advanced metabolic modeling. misosoup helps scientists discover how microbes cooperate by sharing nutrients, a process known as metabolic cross-feeding. When tested on sets of species from different origins, the tool showed that species could thrive in more environments when part of a group. This finding highlights the importance of cooperation in microbial life. misosoup not only predicts these interactions but also provides detailed insights that can guide ecological studies and biotechnological innovation. By revealing how microbes support each other, misosoup contributes to a deeper understanding of life’s interconnectedness and offers tools for solving real-world challenges.

## INTRODUCTION

Microorganisms in nature form highly diverse communities shaped by intricate biotic interactions. While community members often compete for space and resources, they also depend on one another for essential metabolites, such as signaling molecules, siderophores, amino acids, vitamins, and for mitigating the effects of toxic compounds (1). Microbial communities also play pivotal roles in biotechnological applications, including bioremediation, biofuel production, and pollutant biotransformation. Identifying the necessary and sufficient partners that enable species to coexist within these communities is essential for both ecological insight and the development of simplified synthetic communities.

Simplified synthetic communities (generically termed consortia or SynComs) open new opportunities to handle experimentally tractable systems for the study of microbial interactions and the design of microbial communities delivering specific services. Different strategies have been proposed to build these communities, with the “bottom-up” approach—in which isolated strains are combined to build the consortia—being the most prevalent (2–4). The “bottom-up” approach, however, faces some challenges. It requires the prior isolation of each strain, restricting the experiment to culturable organisms only (5). In addition, the number of possible community combinations increases exponentially with the number of strains, making it impractical to culture all potential communities. Therefore, a critical challenge in constructing novel consortia lies in identifying stable combinations of strains that can coexist within a given medium. While recent advancements, such as the development of innovative co-culture plates (6), microfluidic chips (7), and cost-effective protocols for assembling combinatorially complete communities (8), have significantly expanded experimental capabilities, these approaches still face the inherent limitations of traditional “bottom-up” methodologies.

A powerful alternative approach to overcome these limitations comes from computational modeling. In particular, since a significant fraction of microbial interactions are of metabolic nature, metabolic modeling stands out as a valuable complementary approach for the study of microbial communities. This computational framework leverages genome-scale metabolic models (GEMs) of species that encompass all known metabolic reactions within an organism. Furthermore, constraint-based methods, such as flux balance analysis (FBA) (9), enable the determination of growth rates and consumption/secretion patterns of strains at steady state within defined environments. This framework has proven effective in various applications involving clonal populations, including growth prediction, by-product secretion (10), the effect of gene deletions (11), or adaptive evolution (12).

In the context of community-level metabolic modeling, a focus on the search and design of consortia performing specific functions is prevalent, reviewed in references 13–15. An example of this approach is FLYCOP (16), a tool that integrates COMETS (17) to explore multiple community configurations, in order to find the community that optimizes a specified goal. Another example is MinMicrobiome (18), which uses a top-down approach to identify minimal microbial communities capable of performing a specific function, particularly suited for optimizing short-chain fatty acid secretion from AGORA metabolic models. Given the limitations of constraint-based metabolic modeling to handle a large number of strains, graph-theoretic approaches are proposed. For example, CoMiDA (19) finds the smallest set of microbial strains that collectively possess the enzymatic capacity to synthesize a desired target product, a strategy later followed in Metage2Metabo (20).

In addition, metabolic modeling has proven to be a powerful tool in studying microbial ecology. Both constraint-based and graph-theoretic models have been employed to explore ecological interactions (21–23). An important step toward community-level modeling was the work of Klitgord and Segré, who developed an algorithm to find the minimal medium in which pairs of strains could coexist (24). This work inspired other constraint-based methods that extended this search to larger communities (25) and later to evaluate the potential for cooperation and competition of specific consortia implemented in SMETANA (26). These tools facilitate the exploration of broad questions such as the relative importance of cooperation and competition in terrestrial (27) and marine systems (28) or the development of an atlas of putative costless secretions (29).

In this work, we contribute toward the development of community-level metabolic modeling by focusing on the problem of species coexistence. We ask which minimal communities can coexist given a set of strains and a predefined medium. A minimal community is one in which every strain is necessary and sufficient for the survival of all members in the community. We differentiate between complete minimal supplying communities (CMSCs), in which no subset of species in the community can thrive in that medium, and minimal supplying communities (MSCs), which are solutions associated with a particular focal strain. Here, the focal strain is defined as a strain that cannot grow on the selected medium in isolation and therefore requires metabolite supply from partner strains to succeed. In an MSC, although the focal strain depends on its suppliers for growth, subsets of the supplying partner strains may still be able to grow independently in the medium. Therefore, a CMSC is a particular case in which, if any of its members is considered the focal strain, the CMSC itself must appear as one of the corresponding MSC solutions—hence the term “complete.”

**Fig 1:**
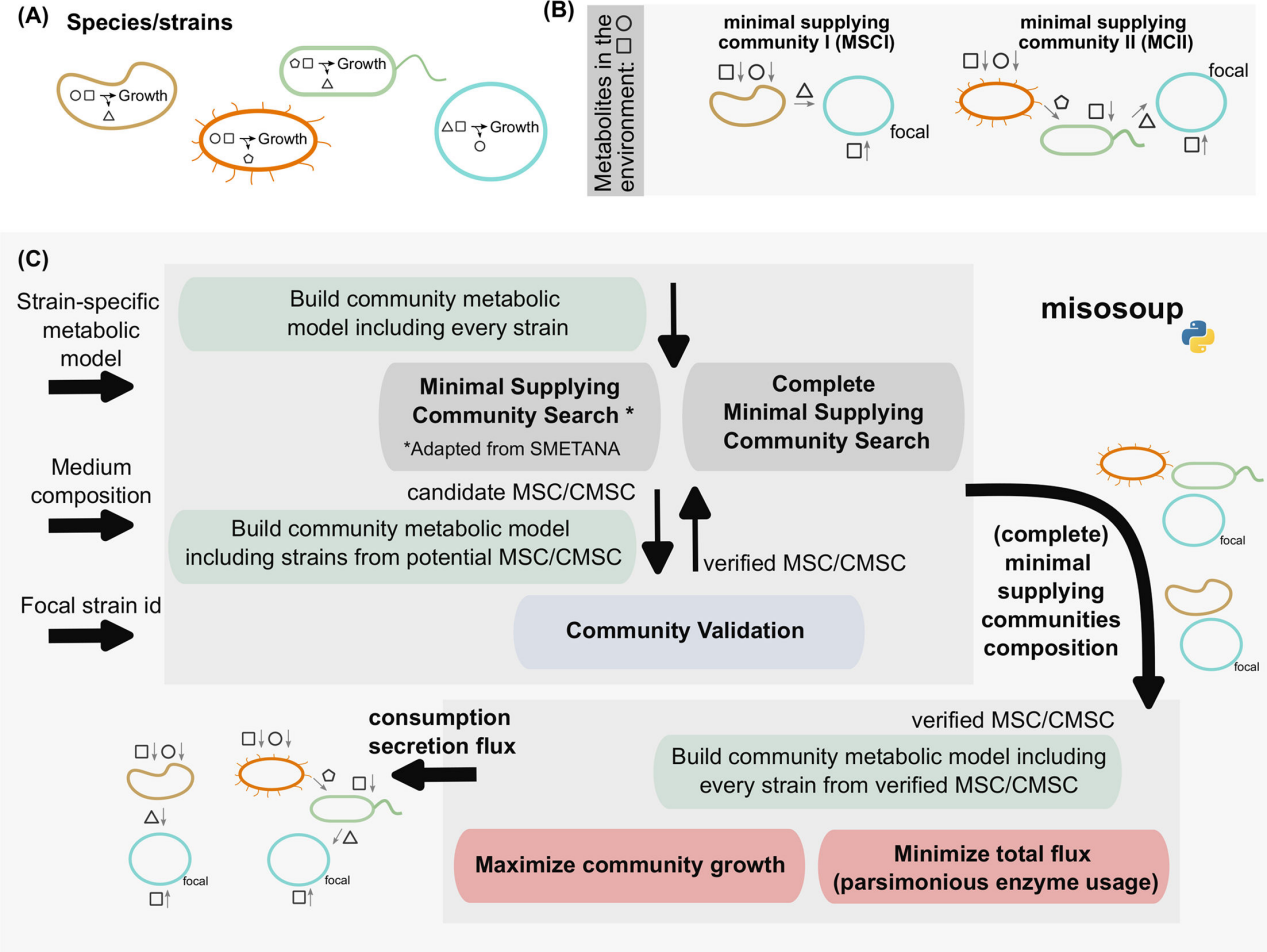
Minimal supplying communities and misosoup. (**A**) Representation of hypothetical bacterial strains with shapes representing metabolic requirements and secreted metabolites. (**B**) Alternative MSCs (I and II) for these strains in an environment with two resources (square and circle), where the blue strain is focal. MSCI and MSCII are minimal as removal of any strain leads to the focal strain’s extinction. (**C**) Explanation of misosoup, its inputs and outputs. The identification process involves two consecutive optimizations: the first step solves a mixed-integer linear programming (MILP) problem to find candidate MSCs, and the second step uses linear programming (LP) to verify that the identified communities satisfy all imposed constraints. Once community feasibility is tested, different optimizations are available to find the consumption and secretion fluxes of every community member.

To address this question, we built on top of methods established in SMETANA to develop an algorithm for the minimal supplying community search (misosoup), implemented as a Python package. A schematic representation of the problem addressed, and its implementation, is presented in Fig. 1. Unlike other methods focusing on finding a minimal community with a prespecified function (e.g., MinMicrobiome) or the potential metabolic exchanges of a specific community (SMETANA), misosoup solves a mixed-integer linear programming (MILP) problem to enumerate all feasible minimal communities for a given set of strains and a specific medium.

Crucially, misosoup adds an evaluation step using linear programming (LP) where every minimal community identified is tested to exclude solutions affected by numerical instability, inherent to complex optimizations. In addition, misosoup also offers optional optimizations, such as maximizing community biomass or minimizing total flux, which provide detailed consumption and secretion rates for each strain in the community.

We illustrate the capacity of misosoup by considering different examples and qualities of metabolic models. First, we tested misosoup on a set of experimentally verified minimal communities with curated metabolic models, finding that it accurately predicts the strains coexisting under the conditions defined experimentally (21). Next, we considered a set of custom metabolic models to show that misosoup can find MSCs of biotechnological interest.

To continue, we studied the ability of misosoup to provide insights into the capacity of predicting growth of auxotrophic strains in coculture experiments (30), with semi-curated AGORA models, a strategy that could be used to grow isolates in media in which they cannot grow. We finally move on to microbial ecology, considering a larger set of strains and draft metabolic models to explore broader questions, such as the prevalence of cross-feeding-driven niche expansion or the possibility of finding functional groups as building blocks of microbial biodiversity (31, 32).

In summary, misosoup is a versatile tool that can be used to aid in organism cultivation, the design of microbial communities, and we demonstrate its applicability to microbial ecology. Software and documentation of misosoup are freely available at https://sirno.github.io/misosoup.

## RESULTS

### misosoup correctly predicts experimentally verified minimal communities

misosoup is designed for large-scale community searches involving many strains and/or media conditions. However, experimentally verified consortia for which curated metabolic models are available remain scarce in the literature, limiting the availability of suitable ground truth data sets. For this reason, and as a proof of concept, we first evaluated a small set of experimentally validated two- and three-strain communities. Although this is far below the scale for which misosoup is intended, these consortia provide reliable validation cases.

**Fig 2:**
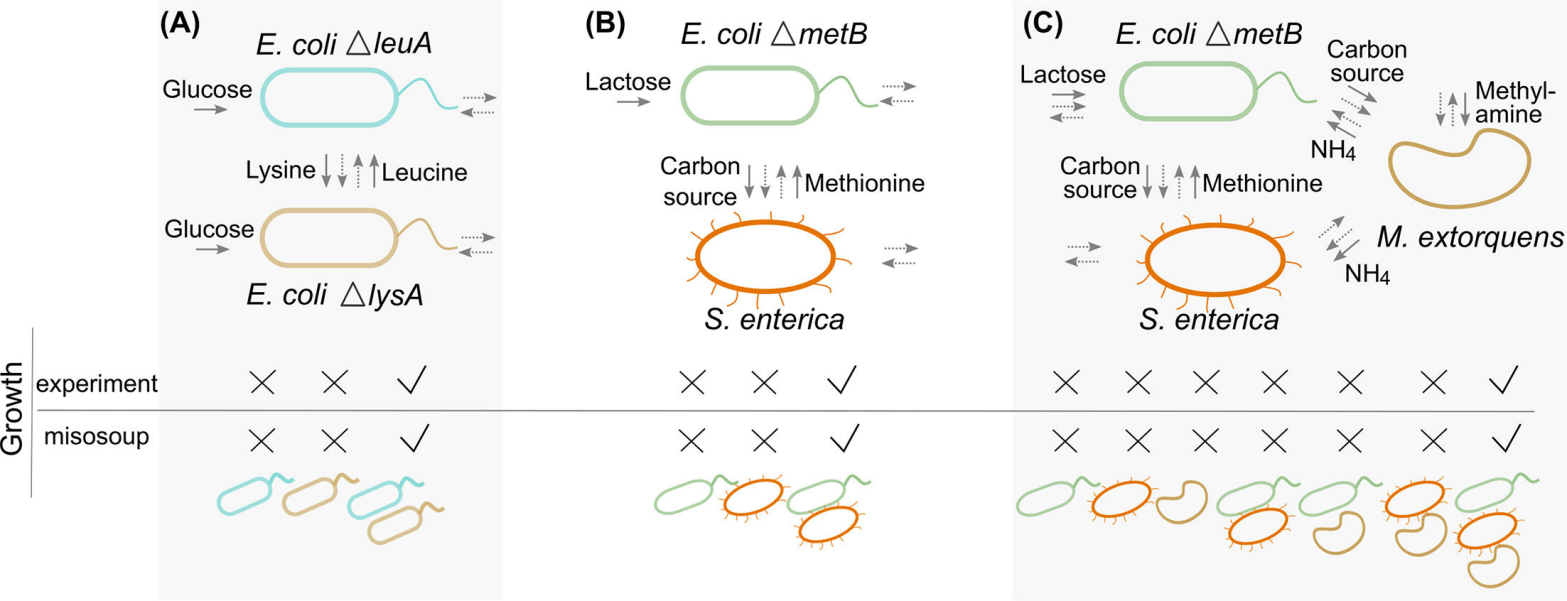
(**A**) Computationally predicted and experimentally tested minimal communities. The strains’ consumption/secretion pattern, as predicted with misosoup, is indicated with arrows: main consumed/secreted metabolites are indicated with arrows and the metabolite name; dashed arrows indicate that misosoup also predicts the consumption/secretion of other metabolites. misosoup correctly predicts that two (**A and B**) or three (**C**) strains are required for growth in glucose minimal medium (**A**), lactose minimal medium (**B**), and lactose minimal medium with methylamine as nitrogen source (**C**).

The first community (Fig. 2A) consisted of two mutant *Escherichia coli* strains auxotrophic for leucine (*E. coli* Δ*leuA*) and lysine (*E. coli* Δ*lysA*), respectively. In glucose minimal medium, the strains grow only when they exchange the essential metabolites leucine and lysine (33). misosoup was able to predict the coexistence of both strains when they were grown in coculture, and their inability to grow in isolation. Moreover, the exchanges found were consistent with the expectation, with *E. coli* Δ*leuA* secreting lysine that is consumed by *E. coli* Δ*lysA*, and the other way around for leucine.

The second community (Fig. 2B) consisted of a methionine auxotroph mutant *E. coli* Δ*metB*, and *Salmonella enterica* mutant strain able to secrete methionine. *S. enterica* cannot grow in lactose, hence relying on the *E. coli* strain (21). misosoup correctly predicted coexistence in lactose minimal medium when ammonium was the nitrogen source, further confirming that both strains were unable to grow independently. The exchange of alternative C-sources consumed by *S. enterica* and methionine required by *E. coli* were also correctly predicted.

In addition, we verified that, when ammonium was substituted by methylamine as nitrogen source, both strains were unable to grow in co-culture, consistent with experiments (21). Following experimental results, we asked if it is therefore possible to create a three-strain obligatory mutualistic consortium by introducing a strain able to assimilate methylamine as a nitrogen source. *Methylobacterium extorquens* is able to use methylamine as both a nitrogen and carbon source. A mutant lacking hydroxypyruvate reductase (Δ*hprA*) cannot assimilate carbon from methylamine, relying on the acetate excreted by *E. coli* for growth, and can provide nitrogen to the other two strains.

misosoup correctly predicted that no pairwise combination of these three strains had positive growth on lactose minimal medium with methylamine as a nitrogen source, while coexistence was only observed when the three strains were grown together (Fig. 2C). Notably, it also correctly predicted the experimentally observed exchanges (21) as detailed in Fig. 2C.

### misosoup finds minimal communities with biotechnological potential

As a first application of misosoup, we considered finding consortia with biotechnological interest, since an important bottleneck is often finding feasible consortia candidates, a question for which misosoup is especially well suited.

To illustrate this application, we searched for consortia able to produce biogas, considering nine strains previously used in the publication of the metabolic modeling tool RedCom (34), whose custom metabolic models were available from the publication. Two of the strains were methanogenic archaea (*Methanospirillum hungatei* [MH] and *Methanosarcina barkeri* [MB]), and seven primary and secondary fermenters (*Escherichia coli* [EC], *Clostridium acetobutylicum* [CA], *Propionibacterium freudenreichii* [PF], *Acetobacterium woodii* [AW], *Desulfovibrio vulgaris* [DV], *Syntrophomonas wolfei* [SW], and *Syntrophobacter fumaroxidans* [SF]) that may generate different compounds from an initial resource such as glucose or ethanol (e.g., acetate and formate) for downstream production of CH_4_ by the archaea.

**Fig 3:**
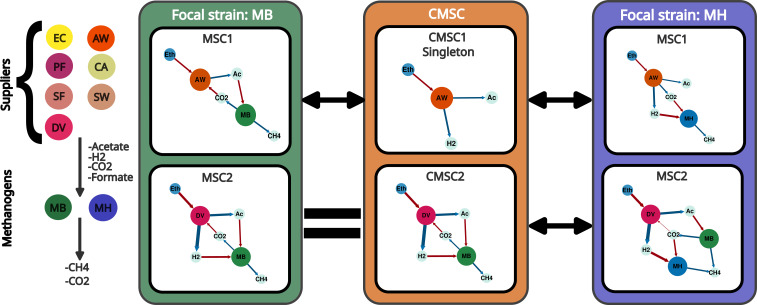
Prediction of biogas-producing consortia. We considered nine strains to identify consortia capable of producing CH_4_ from ethanol. Our analysis yielded two candidate minimal consortia (CMSCs; middle column). One of these consisted of AW alone (a “singleton”). When MB was used as the focal strain, we identified one new consortium and one previously reported CMSC (left column). When MH was used as the focal strain, we found two additional consortia (right column). One of these matches a three-strain consortium described in the literature.

To reproduce results found in reference 34, we focused on minimal communities growing on ethanol as the sole carbon source. We first searched for CMSCs in which no strain can grow in isolation in the specified medium, and whose detection remains elusive for other tools. We found two CMSCs (Fig. 3, middle column); one was a cross-feeding consortium formed by DV and MB, and the other was AW growing in isolation. Note that, although strains growing in isolation do not represent consortia, misosoup returns them since, formally, they fulfill the CMSC definition, which allows the user to identify what strains can grow in isolation. We termed such solutions CMSC singletons.

Since, to produce CH_4_, at least one of the methanogenic archaea is needed, next we searched for MSCs, considering each of the archaea as a focal strain. We found that MB was able to form a cross-feeding consortium with AW and, consistently, the CMSC previously found (Fig. 3, left column). When MH was the focal strain, it also formed a consortium with AW, but there was no supply from MH to AW, as it was the case in the MB-AW consortium, reflecting a commensal structure (Fig. 3, right column). Notably, we found that DV and MB, the second CMSC, also provided the resources needed for MH to thrive, forming a three-strain consortium.

Interestingly, the three-strain consortium found was the one reported in RedCom (34). However, while the authors proposed this consortium because a similar one was previously reported in reference 35, misosoup effortlessly found it as a solution. Importantly, the analysis also provided insights into how the consortium is organized into subunits, with DV and MB forming the core as a CMSC and MH depending on them.

### misosoup predicts large-scale co-culture experiments

The next example considered predicting coculture experiments involving auxotrophic strains that cannot grow in certain media in isolation. We considered an experiment by Oña et al. (30), in which gene deletions were introduced to generate amino acid auxotrophs. The resulting strains were then tested for their ability to grow in coculture, a phenomenon the authors termed “niche expansion.” Therefore, niche expansion here is a situation where an individual organism lacks the capacity for independent growth in a specific abiotic environment but can thrive in that same environment as part of a microbial community.

We selected three strains assayed in the publication (*Bacillus subtilis* 3610, *Escherichia coli* B225113, and *Pseudomonas fluorescens* SBW25) that were available in AGORA2, a database of automatically curated metabolic models (36). For each strain, we generated four auxotrophic metabolic models, each lacking the ability to synthesize one amino acid (leucine, arginine, tryptophan, and histidine corresponding to L, R, W, and H in Fig. 3; see Materials and Methods). We then verified their growth in monoculture across 31 experimental media (see Materials and Methods), each supplemented with the amino acid that the corresponding auxotroph cannot synthesize. Finally, we gapfilled the models whenever growth was observed in supplemented monoculture experiments but not in the models (see Materials and Methods; Supplemental Material). We should note that misosoup’s ability to predict cocultures depends on the experimental criteria considered to determine that growth was observed (e.g., minimum OD or number of replicates in which growth should be observed) because, in turn, these choices determine model gap-filling. In Fig. S1, we evaluated the influence of these choices. In the following, we quantified misosoup’s ability to predict cocultures, restricting the analysis to combinations of media and strains in which individual models of both strains were, after gap-filling, in agreement with the experiments in monoculture.

**Fig 4:**
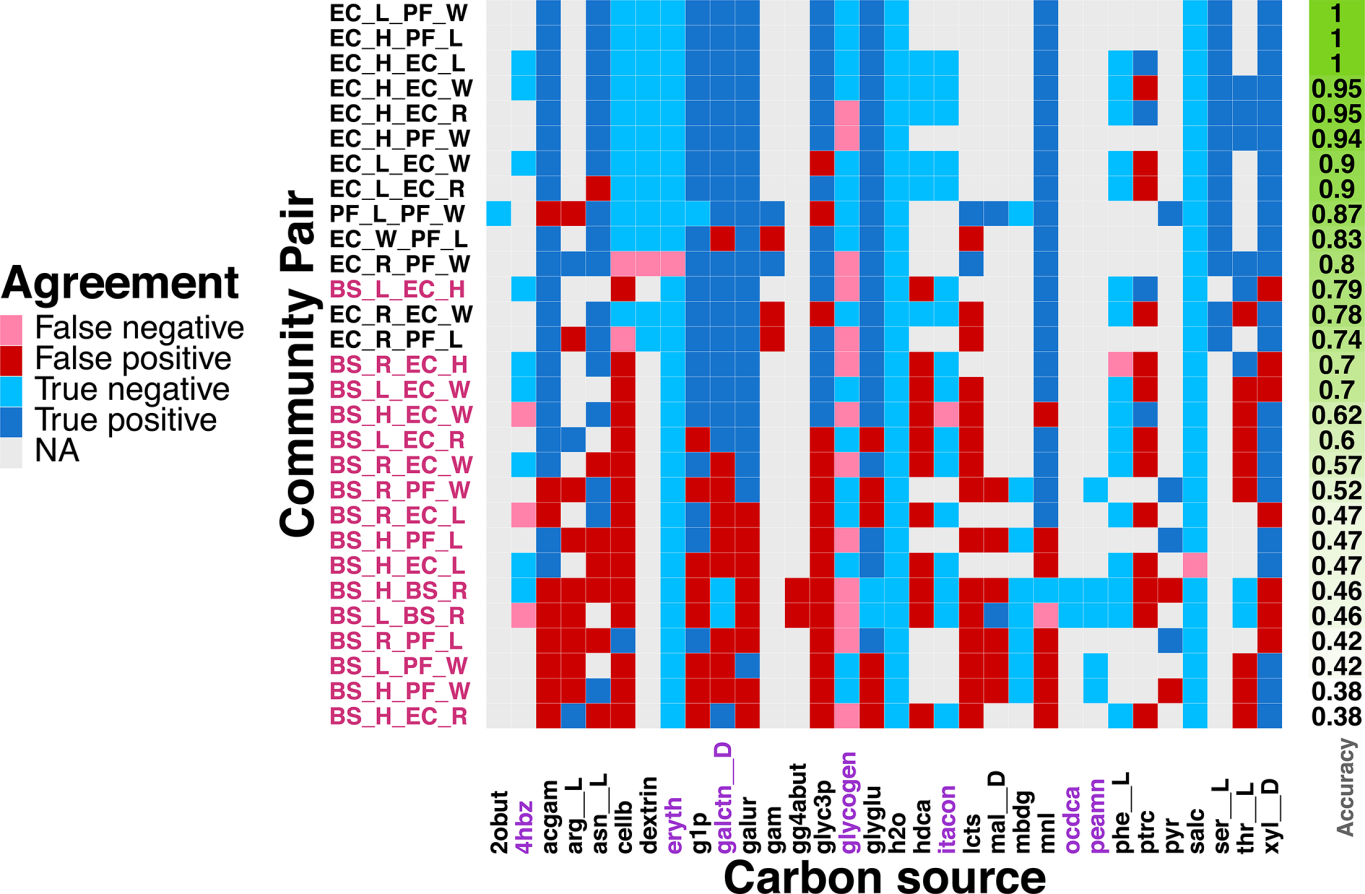
Prediction of coculture experiments. Heatmap representing pairs of auxotrophs cocultured (rows) in different media (columns) with the compound indicated as sole C source. Agreement between misosoup and experiments is shown in blue (true positives and negatives) and disagreements in pink (false positives) and red (false negatives). Gray cells represent experiments in which any of the two members had no agreement with the experiments when grown in monoculture and were excluded from the computation of accuracy (green bar). Labels of co-cultures including a lower quality model (*B. subtilis*) and media that could not be gapfilled are highlighted. These cases explain most discrepancies with model predictions. More details in the text.

In Fig. 4, we show misosoup coculture growth predictions for pairs of auxotrophs grown on different C sources, with blue cells representing agreement between prediction and experiment. Notably, accuracy was above 0.7 in more than half of the experiments performed, highlighting misosoup’s predictive performance.

Importantly, most simulations that did not match the experiment (red/pink cells) are those cocultures in which *B. subtilis* was present (Fig. 4). In Supplemental Material, we show that this model had a poor performance compared to the other two strains, even after gapfilling. Since it tends to generate false positives, it suggests that the model was likely over-gap-filled (see strong increase in sensitivity after gap-filling in Fig. S2). Another source of discrepancy can be attributed to media that could not be gap-filled, which either do not show good agreement in monoculture (e.g., ocdca) or tend to generate false negatives (glycogen).

Finally, misosoup also predicted 25 more cocultures not assayed experimentally, one of them involving three strains (Fig. S5), and even included auxotrophs from the same species (*Pseudomonas fluorescens*), which were not predicted for the other species. We should, however, be cautious with pairs involving *B. subtilis*, since we observed they were often associated with false positives. Our results highlight the potential of misosoup to find conditions in which strains can grow, opening new avenues for culturomics experiments.

### misosoup application in a microbial ecology experiment supports pervasive cross-feeding-driven niche expansion

Next, we illustrate the potential of misosoup to explore broad ecological questions. The strains used in this study were isolated from previous experiments in which natural marine communities assembled on synthetic beads uniformly covered with one or two naturally occurring polymers, mimicking marine snow (37, 38). A reproducible ecological succession was observed to occur on these particles, and it was possible to classify strains as early colonizers, late colonizers, or generalists depending on the stage in which they were observed throughout the succession (38). Interestingly, it was suggested that the capacity of late colonizers to thrive on particles depends on the byproducts produced by early colonizers.

We asked if the succession can be rationalized as an example of niche expansion by investigating a set of 60 marine bacterial strains isolated from these experiments (37, 38), searching for MSCs assayed in 22 different environments. We defined the fundamental niche as the number of environments in which a strain can grow in isolation and the realized niche as the number of environments in which a strain can grow in isolation and in communities. The difference between the realized and fundamental niche constitutes the niche expansion (see Materials and Methods).

**Fig 5:**
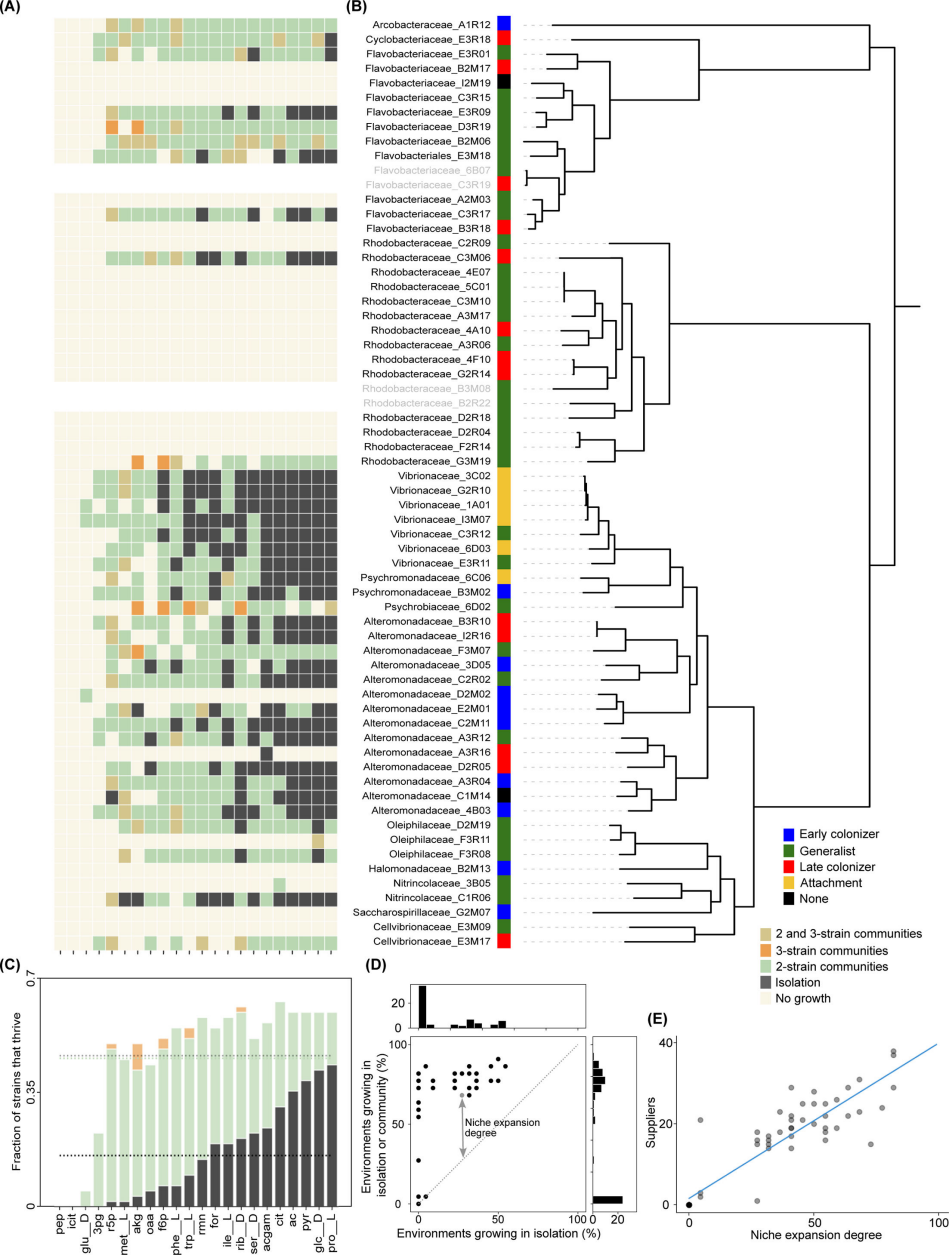
(**A**) Heatmap illustrating whether a strain thrives in a given carbon source when grown in isolation or in communities consisting of two or three strains. Strain names correspond to those shown in the tree in panel **B**, and carbon sources correspond to those in the graph in panel **C**. (**B**) Phylogenetic tree and ecological strategies of the strains, as inferred in reference 38. (**C**) Fraction of strains thriving in each of the 22 tested environments—either in isolation (black bars) or in communities of two (green bars) or three (orange bars) strains. The *x*-axis indicates the short name of the carbon source added to the simulated marine medium (see Supplemental Material for the full names of the metabolites). Dashed lines represent the average fraction of strains thriving in isolation (black), in communities of two strains (green), or in any community size (gray). (**D**) Relationship between the percentage of environments colonized in isolation or communities and those colonized in isolation only. Each dot represents one of the 60 strains. The arrow highlights the degree of niche expansion for the strain shown in gray. Niche expansion is quantified as the difference between environments colonized in communities or in isolation and those colonized in isolation. (**E**) Number of different partners each strain has across all solutions facilitating in which niche expansion was observed and the degree of its niche expansion.

We reconstructed genome-scale draft metabolic models for these strains, using the package CarveMe (39), and simulated growth of each strain in isolation with FBA and in communities with misosoup in 22 different environments (see Materials and Methods). The environments simulated marine broth and differed only in the carbon source available. We selected carbon sources to be carbohydrates, amino acids, or carboxylic acids, in similar proportions (Fig. 5). See Materials and Methods and Supplemental Material for a detailed description of the media composition.

When grown in isolation, we observed significant variation in the fundamental niche sizes of the strains, with closely related strains exhibiting more similar fundamental niches (Fig. 5A and B; Fig. S6). On average, strains were capable of growing in 3.5 environments. Moreover, 32 strains had a fundamental niche size of zero, indicating that they were unable to colonize any of the tested environments (Fig. 5A), with 42% of these strains belonging to the *Rhodobacteraceae* family (Fig. 5B). Notably, we found that fundamental niche size was positively correlated with both the number of genes (Pearson’s *r*^2^ = 0.45) and reactions (*r*^2^ = 0.57; Fig. S7) included in the strain’s metabolic models.

Next, we used misosoup to search for MSCs selecting each strain at a time as focal strain, while the other 59 strains could make up the supplying community. misosoup found a total of 2,210 MSCs. A total of 13% of which were made up of three strains, while the remainder were made up of only two strains. The analysis of these communities shows that the fraction of strains that can colonize an environment increases if we allow strains to assemble into communities. This is true for all environments tested other than those with pep and icit, in which no strains grew in isolation nor in communities.

On average, 10 ± 9 strains (16% ± 14%) grow in an environment in isolation (black line in Fig. 5C), while 29 ± 12 strains (47% ± 19%) grow either in isolation or as part of a community (gray line in Fig. 5C). As a consequence, 41 strains (66%) show some degree of niche expansion, with an average niche expansion degree of 31% (Fig. 5D). This means that strains can colonize, on average, seven more environments in communities than in isolation, and even those strains that colonize a large fraction of niches in isolation exhibit some degree of niche expansion.

Importantly, we found a significant linear relationship between the degree of niche expansion and the number of different partners required for such expansion (Fig. 5E), which means that the more they expand, the more partners they need. Interestingly, the average phylogenetic relatedness of members within the same MSC is above the family level but below the relatedness expected by random pairing (see Fig. S8A), and they also have a significant complementarity in the reactions they host with respect to random pairs (see Fig. S8B and C).

Our analysis identified significant variation in how frequently different strains acted as suppliers within these communities. Consistent with previous observations, the most recurrent suppliers were members of the *Vibrionaceae* and *Alteromonadaceae* families, predominantly associated with the attachment and early colonization stages, respectively (Fig. S9). Notably, these strains primarily supplied resources to members of the *Flavobacteriaceae* family, classified as generalists.

Overall, we see that metabolic interactions between community members make most niches accessible to a larger number of strains. Furthermore, this increased accessibility is aligned with the ecological strategies identified experimentally, with early colonizers facilitating later stages of ecological succession.

### misosoup helps to explore the architecture of microbial biodiversity

Extensive work is needed to elucidate the mechanisms underpinning niche expansion in experimental settings akin to those conducted in previous studies (30, 40). The outputs of misosoup provide insights into the consumption and secretion of individual community members, which may allow us to elucidate the mechanisms underlying niche expansion, and its consequences in biodiversity maintenance.

Given a repertoire of exchanged metabolites, various consumption and secretion rates (referred to hereafter as flux distributions) may still support strains’ growth, posing computational challenges for exploring every alternative leading to niche expansion. Given these complexities, here we concentrate on a single community flux distribution that yielded maximal community growth—the summation of strains’ individual growth rates (see Materials and Methods and Supplemental Material for insights into how other flux distributions could be explored using the output of misosoup).

**Fig 6:**
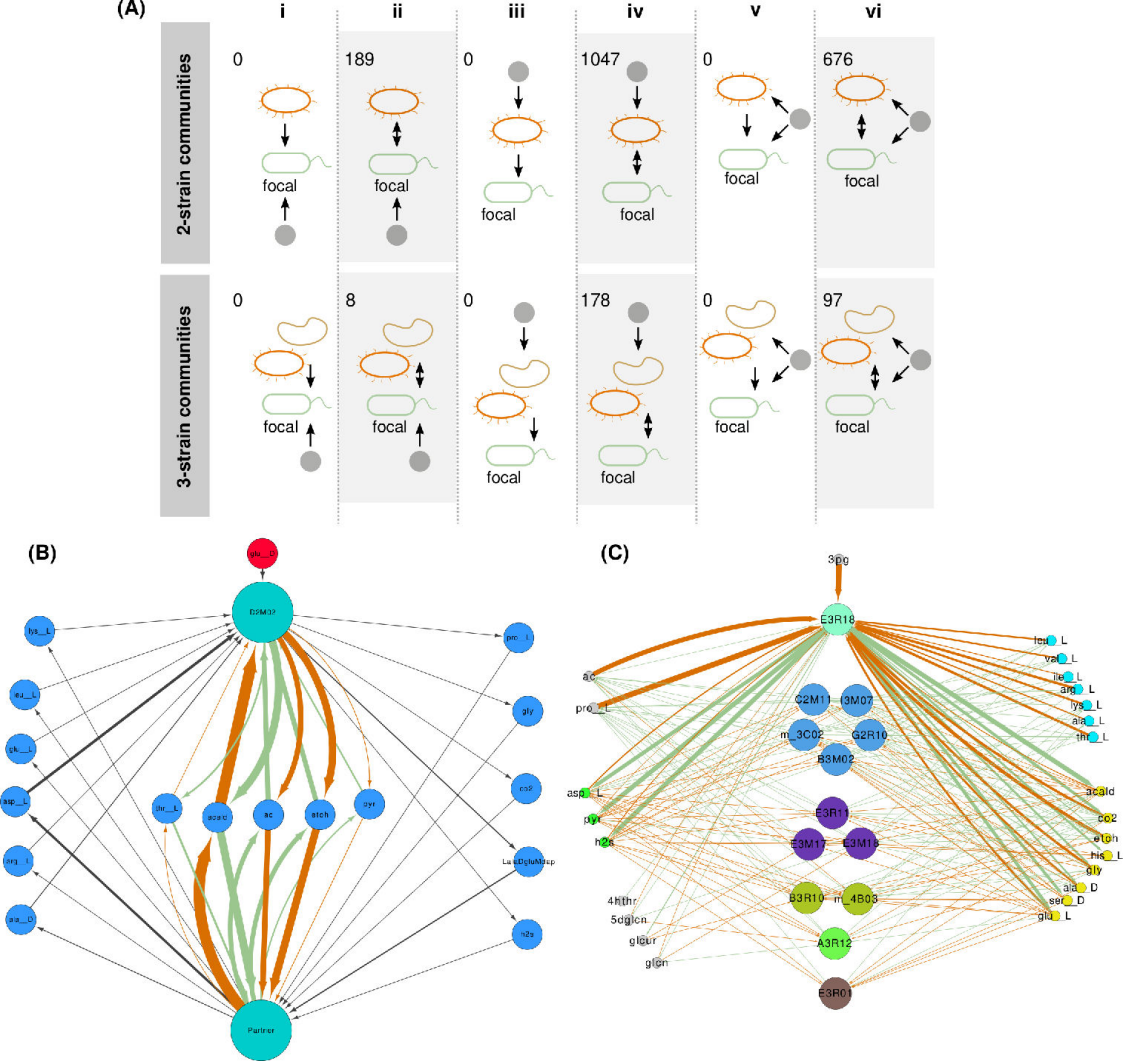
(**A**) Mechanism giving rise to focal strains' niche expansion in the MSCs. The diagram shows whether communities were made up of two or three strains, and whether they belonged to one of six categories: (i) unidirectional cross-feeding where the focal strain consumes the carbon source; (ii) bidirectional cross-feeding where the focal strain consumes the carbon source; (iii) unidirectional cross-feeding where a supplying strain consumes the carbon source; (iv) bidirectional cross-feeding where a supplying strain consumes the carbon source; (v) unidirectional cross-feeding where both strains share the carbon source, or (vi) bidirectional cross-feeding where both strains share the carbon source. Numbers on the top left of each panel indicate the number of communities found in each category. (**B**) The two CMSCs found in glu__D with common links in black and links specific to each solution highlighted in orange and green. Width is proportional to the predicted flux, with those in black selected randomly from either solution. (**C**) The 12 CMSCs solutions found in 3-phospho-D-glycerate (3pg) aggregated. Strains and metabolites are located close in space and with the same color if they belong to the same FG (see the text for details). Orange (green) links represent consumption (secretion).

We investigated the mechanisms giving rise to niche expansion in the focal strains of MSCs. To achieve this, we examined how focal strains and their supplier partners utilized the available carbon source. To this end, we categorized each community flux distribution into six distinct classes (Fig. 6A): (i and ii) the focal strain consumes the carbon source; (iii and iv) a supplying strain consumes the carbon source; or (v and vi) both strains share the carbon source. Additionally, we assessed whether the focal strain secreted metabolites subsequently consumed by supplying strains (ii, iv, and vi) or not (i, iii, and v).

In all MSCs, we observed mutual cross-feeding interactions (ii, iv, and vi in Fig. 6A), where the focal strain consumed metabolites secreted by supplying strains and reciprocally secreted metabolites utilized by those supplying strains. Notably, in the majority of communities (56%), the carbon source was consumed by one of the supplying strains, while in 35% of communities, both the focal and supplying strains shared the carbon source. Note that, since the majority of these MSCs were not complete, the supplier’s consumption of metabolites excreted by the focal strain was not a requirement. In the remaining 9% of communities, only the focal strain utilized the carbon source. We did not detect significant differences in these patterns between supplying communities comprising two or three strains.

Next, to illustrate the potential of misosoup to explore the architecture of these communities, we investigated two sets of CMSCs found on D-glutamate (glu__D) and 3-phospho-D-glycerate (3pg) with two and 12 CMSCs, respectively, all of which were composed of two strains. Exchanged metabolites should be interpreted with caution in these examples since, contrary to other examples presented in previous sections, the presented solutions were obtained with draft metabolic models, and because the problem of finding feasible solutions is highly degenerated. As noted above, more biologically and biotechnologically relevant results often rely on setting realistic flux boundaries.

For the two CMSCs identified on glu__D, strain *Alteromonadaceae*_D2M02 was a common member in both solutions. This is due to *Alteromonadaceae*_D2M02 being the only strain capable of metabolizing glu__D, even though it cannot grow independently and requires a partner. In Fig. 6B, we show the union of both CMSCs with the two partners superimposed in a single node. Both solutions exhibit a shared set of metabolites that are consistently consumed and secreted (indicated by black edges). However, a notable observation is a subset of metabolites exhibiting “orthogonal” secretion and consumption patterns: a metabolite consumed by one partner in one solution (orange edges) is precisely what is secreted by the other partner in the alternative solution (green edges). From *Alteromonadaceae*_D2M02’s perspective, this implies that depending on the specific partner, it can either secrete or consume this particular subset of metabolites, while its interactions with all other metabolites remain consistent across partners.

We also represent the union of 12 CMSCs found on 3pg (Fig. 6C), where *Cyclobacteriaceae*_E3R18 (top) appears in all solutions. In this case, *Cyclobacteriaceae*_E3R18 and another strain (*Cellvibrionaceae*_E3M09) are the only two strains that can take up 3pg. We classified strains and metabolites into functional groups using functionink (41). In short, the method classifies strains as belonging to the same functional group if they consume and secrete similar metabolites in their respective solutions (see Materials and Methods).

The analysis revealed five different functional groups, all of them supplying acetate and proline, that were consumed by *Cyclobacteriaceae*_E3R18, except for Flavobacteriaceae_E3R01, which only secreted proline (central nodes in Fig. 6C). Interestingly, each group has a distinctive set of amino acids secreted (cyan metabolites, top right) that were consumed by *Cyclobacteriaceae*_E3R18. Following groups in the network from top to bottom, the amino acids were Leu, Val, Arg, Thr (blue), Leu, Arg, Lys (violet), Ala_L (light green), Lys, Ala (olive), and Lys (maroon). In turn, they uptake different compounds secreted by *Cyclobacteriaceae*_E3R18 (pink nodes), among which we find Gly, Ala_D, Ser, and Glu. Bearing in mind the caveats noted above on the interpretation of the fluxes, it is in principle possible to identify interchangeable strains and their capacities, while also identifying the key metabolites that need to be present in the medium. This opens opportunities to learn how to build new communities or the supplements that a specific strain may need to thrive.

## DISCUSSION

In this study, we introduce misosoup, a Python package developed to identify the necessary and sufficient partner strains that enable microbial survival in defined environments—referred to as MSCs and CMSCs (see Fig. 1).

misosoup builds upon methods used in the SMETANA package (26), which was designed to analyze metabolic interaction potentials between strains, summarized as part of the SMETANA score, which aggregates communities and metabolic exchanges. In contrast, misosoup focuses on identifying minimal communities that support the growth of all strains in the community. This difference in focus is reflected in the outputs of the two packages and poses additional challenges for misosoup in terms of computational complexity. As the communities misosoup identifies are used for further analysis, the communities need to be verified to ensure that they do not rely on numerical instability or other artifacts that may arise during the optimization process. This ensures that the communities identified by misosoup are biologically meaningful and will be reported consistently. To highlight the complementarity of these packages, we simulated a few examples using both tools (see Supplemental Material).

Several other relevant packages and algorithms exist, each designed for specific purposes. However, misosoup is distinct in several key ways. First, the minimal communities identified by misosoup are defined by their ability to support strains’ coexistence, rather than being optimized for a purpose such as the synthesis of specific product metabolites (16, 18–20, 42). Second, misosoup specifically searches for the necessary and sufficient community members required for strains’ coexistence, rather than focusing on minimizing the number of strains in a community (19, 43)—two different concepts often referred to as “minimal communities.” Third, misosoup employs constraint-based methods, unlike graph-theoretic approaches (19, 20, 42, 43), which do not require biomass reactions or precise stoichiometry. This constraint-based approach enhances the accuracy of its predictions.

We explored the potential of misosoup to investigate potential applications in biotechnology by first focusing on biogas-producing consortia, finding all MSCs that could grow on ethanol, including those that produced CH4. Notably, we found a previously reported three-strain consortium, and we illustrated how finding CMSCs allowed us to dig into the modular structure of this consortium. As a limitation, misosoup is not designed to search for large communities or to analyze specific communities, for example, optimizing the production of a specific compound, for which other tools are available (16, 34). Instead, misosoup is intended to search MSCs for large sets of strains and media, facilitating the exploration of potential consortia and providing guidance on potential assembly rules (e.g., starting from CMSCs and incorporating other strains on top of them).

In another example, we reproduced results from coculture experiments in which pervasive niche expansion was observed (30). We found accuracies well above 0.7 for half of the pairs explored and predicted new ones. There is growing interest in this strategy as shown in another study, where the authors identified 125 instances in which a particular carbon source did not support strain growth in monoculture, but only 21 cases where co-culture also failed to support growth (40). Thus, the occurrence of no growth in co-culture dropped to 17%, highlighting the potential of this strategy for culturomics.

We also explored the potential of misosoup to investigate ecological questions focusing on niche expansion on a larger set of marine strains (44) isolated from experiments of ecological succession of natural communities assembling under controlled conditions (37, 45). Interestingly, our results are consistent with the ecological strategies identified for these strains, with those found in earlier stages (interpreted in *in vitro* experiments with the same strains as degraders and exploiters [46]) being more often suppliers of strains with smaller fundamental niches, which are late colonizers in the experiments. The relationship between facilitation and niche expansion has been previously observed in large-scale studies showing that it may be responsible for bacterial cosmopolitanism (47) and community-level coexistence (48). Although we did not explicitly explore this question, we acknowledge there is growing interest in the evolutionary consequences of the prevalence of facilitation summarized under the black queen hypothesis (49), boosting efforts toward the search for auxotrophies (1, 48, 50) and their role in stability (51).

The importance of facilitation seems to contrast with classical niche theory, which primarily focused on negative biotic interactions, such as predation and competition for space and resources, which constrain an organism’s fundamental niche (52, 53). In the last two decades, however, there was a shift in focus in macroscopic ecology from the study of competitive exclusion conditions to coexistence conditions (54, 55), highlighting the positive role of mutualistic interactions in promoting stability (56, 57), by reducing the effective competition that species experience (58, 59).

In the microscopic world, there is also growing evidence pointing toward the importance of cross-feeding in structuring communities (60). As a consequence, a large body of theoretical research investigates the role of cross-feeding in microbial community stability (44, 61), although with fewer examples showing community-level interaction structures that facilitate coexistence (32, 62, 63). We illustrated how misosoup facilitates the large-scale analysis of communities, finding that most cross-feeding interactions were bidirectional.

One reason limiting research in this direction beyond toy models is the computational burden associated with searching and analyzing communities built with metabolic models. In contrast, our approach can, in principle, uncover communities of any size. This capability is due to misosoup’s ability to identify minimal communities without exhaustively testing all possible community combinations. For example, with a data set of 60 strains (as used in this study), there are 1,770 possible two-strain communities, 34,220 three-strain communities, and so on, making exhaustive testing impractical if not impossible.

We showed that misosoup may help to explore broad theoretical questions such as biodiversity organization, also with potential biotechnological impact. For instance, if there is no need to interact with multiple partners to thrive in a given medium, questions such as how large multispecies communities are sustained remain open. This question connects with how common functional redundancy is in microbial communities, a topic attracting high interest in the field (64). Thanks to the accessible output provided by misosoup, it is straightforward to identify functional groups with existing tools (41) by searching for strains consuming and secreting similar metabolites. Identifying strains that may be interchangeable under certain conditions may be invaluable to design new communities and to understand long-standing questions such as how biodiversity is organized from functional groups or how they may combine to build cohesive consortia (31).

Our work has limitations. First, we showed that misosoup’s predictive power depends on the quality of the metabolic models. We started with a proof-of-concept example with curated models, then considered semi-curated models for coculture experiments, followed by custom models in the biogas example, and ending with draft metabolic models for the marine communities. Despite misosoup’s ability to handle different models, the lower the quality, the less reliable the predictions are and the conclusions drawn from them. Therefore, for broad ecological questions such as the extensive niche expansion observed, it remains to be seen whether it is specific to these strains or if it represents a general property of organisms. Although our results with more reliable models also point toward the same conclusion, future studies could replicate our analysis with different sets of species or with random viable organisms that emulate genome-scale metabolic models but contain a random complement of biochemical reactions drawn from a “universe” of such reactions (65).

Second, our insights on the mechanism driving niche expansion come from analyzing a community flux distribution compatible with maximal community growth. However, other objectives (66, 67) might better represent species growth in communities. Exploring these alternatives is straightforward within our framework. misosoup can identify the minimal communities (which are not affected by this optimization criterion), and other packages can then be used to find flux distributions aligned with other objectives of interest. In Supplemental Material, we provide an example of how to export misosoup results.

Third, despite having the capacity to find communities of any size, the vast majority of communities we found had only two members. This could be the consequence of several technical factors specific to our analysis, such as the use of simple media composition, the use of draft metabolic models that could be over-annotated (68), or the number of available strains. Therefore, it remains to be investigated how our results are affected by the total number of strains included in the analysis or the complexity of the medium. As the number of strains increases, so does the potential for niche expansion, as the likelihood of including a strain with the right set of reactions to complement another one increases. On the other hand, more recalcitrant media may lead to larger minimal communities because more trophic levels may be needed, as described in reference 46. The interplay between both, and how large communities are built from minimal ones, remains an open question.

In summary, we developed a package to identify minimal communities, allowing us to explore how communities of any size (not just strain pairs) can expand a strain’s niche, enabling different potential applications. We hope our tool may contribute to investigating broad ecological questions built around species coexistence and the development of biotechnological research.

## MATERIALS AND METHODS

### Metabolic models

In the following, we briefly describe the models used, with details regarding the generation of mutants and gapfilling procedures available in Supplemental Material.

First, we tested misosoup by performing three analyses with 2 two-strain and 1 three-strain consortia, which were experimentally identified to make up minimal communities (21, 33). For the first analysis with two mutant *E. coli* strains auxotrophic for leucine and lysine (33), we used *E. coli*’s metabolic model *i*JO1366 (69), which we downloaded from the BIGG database (70). For the other two analyses, we used the metabolic models of *E. coli*, *S. enterica*, and *M. extorquens* AM1 used in reference 21 that were provided to us by the authors. For consistency between models, we modified the identifiers of metabolites and exchange reactions (see Supplemental Material).

For predictions of coculture experiments, we considered for simulation three out of five strains assayed in the experiments we attempted to reproduce (30), because their metabolic models were available in the AGORA2 database (36): *Bacillus subtilis* 3610, *Escherichia coli* B225113, and *Pseudomonas fluorescens* SBW25. We introduced in each model a metabolite ID and exchange reaction for each of the 31 carbon sources experimentally assayed, and gapfilled the models to grow on MMAB supplemented with acgam (N-acetyl-D-glucosamine), because this was the medium they used experimentally to test that all strains were able to grow in monoculture. Further gapfilling was needed for specific carbon sources depending on experimental criteria to determine that growth was observed, detailed in the Supplemental Material. For consistency, in our text, we chose the same criteria used by the authors in reference 30.

To simulate biogas-producing communities, the GEMs were directly retrieved from the RedCom publication (34). All the models follow the same custom namespace and were not modified.

Finally, we used CarveMe (39) to reconstruct the metabolic models for 60 marine strains for which whole-genome data were available with standard parameters. We simulated marine broth conditions. The detailed composition of the media and the 22 carbon sources selected are available in the Supplemental Material.

### Flux balance analysis

FBA is a method to predict metabolic flux—the rate at which chemical reactions convert substrates into products—through every reaction in a metabolic model (9, 10). It is based on two central assumptions: first, that cells optimize some metabolic property such as the growth rate (equal to the flux of an artificial biomass reaction); and second, that cells are in metabolic steady-state.

To compute the value of that objective metabolic output, FBA relies on the stoichiometry of every reaction involved in the process. This is mathematically represented as the stoichiometric matrix S of size m×v, where m denotes the number of metabolites, and v denotes the number of reactions in the model. Each element $S_{ij}$ corresponds to the stoichiometric coefficient with which metabolite i participates in reaction j. Additional constraints can be added by setting lower ($l_{j}$) and upper ($u_{j}$) bounds on the reaction flux ($r_{j}$). All variables and parameters are summarized in Table 1. The optimization problem that FBA solves can be written as a linear programming problem:


(1) $$\begin{array}{ll} \text{maximize} & r_{g}\\ \text{subject to} & Sr=0\\ & l_{j}\leq r_{j}\leq u_{j} \end{array}$$

**TABLE 1:** Definitions for the algorithmic MILP/LP problems

Variable	Definition
$y_{i}\in \{0,1\}$	Binary values determining active/inactive strains
S	Strain stoichiometric matrix
$\hat{S}$	Community stoichiometric matrix
$r\in R^{k}$	Reaction rates
$l,u\in R^{k}$	Lower and upper bounds
$r_{j}^{\left(i\right)}\in R$	Reaction associated with strain i
$r_{g}^{\left(i\right)}\in R$	Biomass reaction of strain i
a,b∈R	Bounds for growth constraints
G	Set of strains
C	Candidate community
$E\subset 2^{G}$	Solutions, each solution E∈E has size |E|
$K\subset 2^{S}$	Excluded solutions, each solution K∈K has size |K|
S∖K	Complement of solution K (inactive strains in that solution, with size |G|−|K|)

We performed FBA on single strains with COBRApy (71).

### Minimal community search

We find minimal communities by searching a larger community metabolic model for minimal sets of strains that can sustain growth. The community metabolic model is constructed by combining all individual metabolic models of the organisms into a single metabolic model with a joint stoichiometric matrix as in reference 72. This formulation allows each organism to exchange metabolites with the environment and with other community members through a shared interface.

Participation of an organism in the community is modeled using a binary variable ($y_{i}$) as in SMETANA (26). When a strain is active, the binary variable is set to 1. The strain has to fulfill minimal growth requirements ($r_{g}^{i}>a$), and it can exchange metabolites with the environment and other strains. If the binary variable is set to 0, the strain is inactive and cannot exchange metabolites. Note that a key difference with the work of Zelezniak et al. (26) lies in how the active/inactive state of a strain is modeled. In reference 26, the sum of secretions is set to zero when a strain is inactive, while in misosoup, the flux through every reaction is set to zero.

Minimal communities are those that minimize the sum of binary variables. We find these by solving the associated MILP problem, similar to SMETANA, by solving the MILP problem specified in equation 2. This problem is constrained by the stoichiometric matrix of the community ($\hat{S}$) and lower and upper bounds for each reaction ($l_{j},u_{j}$), and bounds on the biomass reaction (a and b). When a strain is inactive, all its reaction bounds are set to zero, forcing all fluxes to zero.

In addition, two types of constraints are added to progressively restrict the search space. For each accepted community E∈ℰ, an integer-cut constraint prevents the identical set of active strains from appearing in subsequent solutions. For each discarded solution K∈𝒦, we define its complement as $𝒮\setminus K=\left\{i\in 𝒮|y_{i}=0\right\}$, i.e., the set of strains that were inactive in that solution. A constraint then forces at least one strain in 𝒮∖K to be activated, ensuring that any subsequent candidate community contains at least one strain absent from K. These constraints correspond to the last two lines of equation 2.


(2) $$\begin{array}{llcl} \text{minimize} & \sum_{i\in S}y_{i} & & \\ \text{subject to} & \hat{S}r=0 & l_{j}\leq r_{j}\leq u_{j} & \\ & r_{j}^{\left(i\right)}-l_{j}^{\left(i\right)}y_{i}\geq 0 & r_{j}^{\left(i\right)}-u_{j}^{\left(i\right)}y_{i}\leq 0 & \\ & r_{g}^{\left(i\right)}-ay_{i}\geq 0 & r_{g}^{\left(i\right)}-by_{i}\leq 0 & \\ & \sum_{i\in E}y_{i}\leq |E|-1 & & \forall \;E\in E\\ & \sum_{i\in S\setminus K}y_{i}\geq 1 & & \forall \;K\in K \end{array}$$

### Minimal community validation

Solutions to the MILP problem represent potential minimal communities; however, some solutions may be affected by numerical instability inherent to complex problems such as the one we are solving (caused by bigM-constraints for binary variables [73]). In order to verify that the found communities indeed satisfy all constraints imposed (and are therefore feasible solutions), we perform a second optimization with the LP specified in equation 3. For this, we build a community model including only those strains found to compose a potential minimal community (${\hat{S}}_{C}$), and we maximize the sum of biomass production ($\sum r_{g}^{i}$) subject to steady-state ($S_{C}r=0$) and constraints on strain’s reaction flux ($r_{j}^{i}-l_{j}^{i}y_{i}\geq 0$ and $r_{j}^{i}-u_{j}^{i}y_{i}\leq 0$) while forcing the strains from the potential community to be in the active state ($r_{g}>a$). If the optimization is successful, we report the community as a minimal community and add it to ℰ. If the optimization is infeasible, we add the potential community to 𝒦, excluding it from further searches.


(3) $$\begin{array}{lll} \text{maximize} & \sum_{i\in C}r_{g}^{\left(i\right)} & \\ \text{subject to} & {\hat{S}}_{C}r=0 & l_{j}^{\left(i\right)}\leq r_{j}^{\left(i\right)}\leq u_{j}^{\left(i\right)} \end{array}.$$

### Niche expansion of marine strains

For each marine strain, we define its fundamental niche (F) as the fraction of environments in which it can thrive in isolation, and its realized niche ($F^{*}$) as the fraction of environments in which it can thrive in isolation or in a community. The niche expansion (ΔF) is defined as the difference between its fundamental niche and its realized niche: $\Delta \;F=F^{*}-F$.

### Strains ecological features, phylogeny, and determination of functional groups

We considered the ecological strategies and phylogeny of marine strains inferred from previous work; we provide a brief description here for completeness. Full details can be found in reference 38. Ecological strategies of strains were inferred by analyzing the dynamical behavior of exact sequence variants (ESVs) in assembly experiments of marine communities on synthetic particles described in above. These experiments had four differentiated compositional stages describing a community-level ecological succession. ESV strategies were defined by associating a significant preference to these stages by performing a zero-inflated negative binomial regression between each ESV abundance and the factor determining the stages. Strains’ strategies were considered those found for ESVs having a perfect match to them in a 16S rRNA gene alignment.

The phylogeny was also built in previous work (38) using GTDB-Tk (74), considering 120 marker genes and formatted in iTOL (75). Phylogenetic distances were computed from the phylogeny obtained with the function cophenetic.phylo of the R package ape (76).

Finally, to determine functional groups in Fig. 6, we aggregated all solutions into a single network and analyzed it with functionink (41). This algorithm quantifies an all-against-all similarity between the nodes of a network, where more similar nodes are those connected with approximately the same neighbors with the same type of link (for instance, two species in the same functional group should consume and secrete similar metabolites). To determine functional groups, nodes are clustered according to their similarity, and we considered the maximum of the total partition density as an automatic stopping criterion to determine the optimal partition (see reference 41 for details).

## Supplementary Material

Reviewer comments

## Data Availability

misosoup is freely available at https://sirno.github.io/misosoup/, and the last release is permanently stored in Zenodo with DOI 10.5281/zenodo.15791905. The 60 genomes of marine isolates are available in NCBI with the BioProject identifiers PRJNA414740 and PRJNA478695. The reconstructed models (in sbml format) and additional data to reproduce results can be found at https://github.com/sirno/niche_expansion_data, stored in Zenodo with DOI 10.5281/zenodo.16744429.

## SUPPLEMENTAL MATERIAL

Supplemental Material msystems.00688-26-s0001.pdf Supplemental materials and methods.

OPEN PEER REVIEW reviewer-comments.pdf An accounting of the reviewer comments and feedback.

ASM does not own the copyrights to Supplemental Material that may be linked to, or accessed through, an article. The authors have granted ASM a non-exclusive, world-wide license to publish the Supplemental Material files. Please contact the corresponding author directly for reuse.
