# Supplementary material for: misosoup: a metabolic modeling tool for identifying minimal microbial communities, facilitates the exploration of microbial ecology and biotechnological applications
Source: mSystems. 2026 Jul 28;11(8):e00688-26. doi: 10.1128/msystems.00688-26 (PMC13483462; doi:10.1128/msystems.00688-26)
Supplement: Supplementary file 1 [file msystems.00688-26-s0001.pdf]

Supplementary Materials

misosoup: A metabolic modeling tool for identifying minimal microbial communities provides valuable insights into microbial ecology and biotechnological applications.

Nicolas Ochsner[1,♮], Magdalena San Román[1,2,♮,*], Adrián Jiménez-Fernández[2], Sebastian Bonhoeffer[1] & Alberto Pascual-García[1,2,*].

(1) Institute of Integrative Biology, ETH-Zurich, Zurich, Switzerland. (2) Centro Nacional de Biotecnología, CSIC, Madrid, Spain. (♮) Equal contribution, order was determined by increasing seniority. (*) Address correspondence to: sanroman.magdalena@gmail.com, alberto.pascual@csic.es.

Contents

1 Supplementary Materials **1**

1.1 Supplementary Methods 1

1.1.1 Modification of identifiers in metabolic models 1

1.1.2 Construction of mutants 2

1.1.3 Detailed composition of media 2

1.1.4 Gapfilling metabolic models for coculture experiments'predictions 6

1.1.5 Working with minimal communities outside misosoup 11

1.2 Supplementary Results 12

1.2.1 Supplementary Note. Comparison of packages SMETANA and misosoup . 12

1.2.2 Supplementary Note. Additional results for coculture experiments 14

1.2.3 Supplementary Note. Additional results for marine strains simulations . . 14

1 Supplementary Materials

1.1 Supplementary Methods

1.1.1 Modification of identifiers in metabolic models

We modified the identifiers of metabolites and exchange reactions only to ensure consistency between models, and they are not required for the use of `misosoup`.

In models used in the first analysis of 2- and 3-species consortia, we replaced every compartment indicator `[e]`, `[p]` and `[c]`; or `(e)`, `(p)` and `(c)` by `_e`, `_p` and `_c` respectively. Further, in *S. enterica*'s model we changed the identifiers of the following external metabolites (<old>/<new>): `cam/cm`, `galctr__D/galct__D`, `glcn__D/glcn`, `metox__R/metsox__R__L`, `metox/metsox__S__L`, and `orn__L/orn`. Similarly, we changed the identifiers of two external metabolites in *M. extorquens*' model: `glcn__D/glcn` and `mea/mma`. Additionally, we changed the biomass reaction identifier from `BIO2b_Mex` to `Me_biomass`. Last, we changed the identifiers of every exchange reaction in all three models so that they were of the form EX_<identifier>. For example, the external reaction corresponding to glucose (with metabolite id `glc__D_e`) is of the form `EX_glc__D_e`.

1.1.2 Construction of mutants

For *E. coli*'s metabolic model *i*JO1366 [1] used in the first example, we modified it to build the respective mutant strains: for mutant lysA, we constrained flux through the associated reaction (DAPDC) to zero; for mutant leuA, we constrained flux through the associated reaction (IPPS) to zero.

In the example predicting coculture experiments, we created models for auxotrophic strains from WT gapfilled models for leucine (L), arginine (R), tryptophan (W) and histidine (H) amino-acids. For this, we blocked in the models every reaction that biosynthesizes the specific amino-acid. We also blocked catabolic pathways using these amino-acids. This means that we set lower and upper bounds to zero for every reaction in which the metabolite was either produced or consumed. We took this rather conservative approach because, experimentally, they used different ways to generate auxotrophies which may not be in reproducible in the models. For instance, Oña's *et al.* [2] knocked out specific genes (argH, leuB, trpB or trpC and hisD) and

Table 1: Metabolite composition of the MMAB base media.

**ID**	**Name**	**Formula/Ion**
ca2	Calcium	Ca^{2+}
cl	Chloride	Cl^{-}
cobalt2	Cobalt	Co^{2+}
cu2	Copper	Cu^{2+}
fe2	Iron (II)	Fe^{2+}
k	Potassium	K^{+}
mg2	Magnesium	Mg^{2+}
mn2	Manganese	Mn^{2+}
na1	Sodium	Na^{+}
nh4	Ammonium	${NH_4}^{+}$
o2	Oxygen	O_2
pi	Phosphate	${PO_4}^{3-}$
so4	Sulfate	${SO_4}^{2-}$
zn2	Zinc	Zn^{2+}
h2o	Water	H_2O
h	Protons	H^{+}

found the desired phenotype, while in the models there were alternative enzymes. Indeed, *E.coli* and *P.fluorescens* WT models were able to grow with the amino-acid as a sole carbon source, a behavior that was not observed in the experiments.

1.1.3 Detailed composition of media

Coculture experiments In coculture experiments, composition of MMAB base media is displayed in Suppl. Table 1, and it was available in unlimited amounts. In addition, each of the 31 C sources considered in biolog plates are shown in Suppl. Table 2.

Marine medium We simulated a marine environment assuming that ammonium, biotin, calcium, chloride, cobalamin, cobalt, copper, folate, iron, magnesium, manganese, molybdate, nickel, nicotinic acid, oxygen, p-aminobenzoic acid, pantothenic acid, phosphate, potassium, protons, pyridoxal, riboflavin, selenate, selenite, sodium, sulfate, thiamine, tungstate, water, and zinc were available in non-limiting amounts.

For each marine environment a single carbon source was available at a time. The 22 carbon sources used in the analysis were selected through a model-based screening approach. First, Flux Balance Analysis (FBA) simulations were performed in marine medium using each potential car-

Biolog ecoplate carbon sources-biggID relation	
Metabolite	BiGG_ID
Water	h2o
β-Methyl-D-Glucoside	mbdg
D-Galactonic Acid γ-Lactone	galctn__D
L-Arginine	arg__L
Pyruvic Acid Methyl Ester	pyr
D-Xylose	xyl__D
D-Galacturonic Acid	galur
L-Asparagine	asn__L
Tween 40	hdca
i-Erythritol	eryth
2-Hydroxy Benzoic Acid	salc
L-Phenylalanine	phe__L
Tween 80	ocdca
D-Mannitol	mnl
4-Hydroxy Benzoic Acid	4hbz
L-Serine	ser__L
α-Cyclodextrin	dextrin
N-Acetyl-Glucosamine	acgam
γ-Amino Butyric Acid	gg4abut
L-Threonine	thr__L
Glycogen	glycogen
D-Glucosamic Acid	gam
Itaconic Acid	itacon
Glycyl-L-Glutamic Acid	glyglu
D-Cellobiose	cellb
Glucose-1-phosphate	g1p
α-Keto Butyric Acid	2obut
Phenylethylamine	peamn
α-D-Lactose	lcts
D,L-α-Glycerol phosphate	glyc3p
D-Malic Acid	mal__D
Putrescine	ptrc

Table 2: Carbon sources of the Biolog Ecoplates and its BIGG ids. In the metabolites which does not have a direct ID, proxy metabolites where selected

Table 3: Carbon sources used in marine environments.

**ID**	**Name**	**Formula**
pep	Phosphoenolpyruvate	$C_3H_2O_6P^{2-}$
icit	Isocitrate	$C_6H_5O_7{}^{3-}$
glu__D	D-Glutamate	$C_5H_8NO_4{}^{-}$
3pg	3-Phospho-D-glycerate	$C_3H_5O_7P^{2-}$
r5p	Ribose 5-phosphate	$C_5H_9O_8P^{2-}$
met__L	L-Methionine	$C_5H_{11}NO_2S$
akg	2-Oxoglutarate	$C_5H_4O_5{}^{2-}$
oaa	Oxaloacetate	$C_4H_2O_5{}^{2-}$
f6p	D-Fructose 6-phosphate	$C_6H_{11}O_9P^{2-}$
phe__L	L-Phenylalanine	$C_9H_{11}NO_2$
trp__L	L-Tryptophan	$C_{11}H_{12}N_2O_2$
rmn	L-Rhamnose	$C_6H_{12}O_5$
for	Formate	$CHO_2{}^{-}$
ile__L	L-Isoleucine	$C_6H_{13}NO_2$
rib__D	D-Ribose	$C_5H_{10}O_5$
ser__D	D-Serine	$C_3H_7NO_3$
acgam	N-Acetyl-D-glucosamine	$C_8H_{15}NO_6$
cit	Citrate	$C_6H_5O_7{}^{3-}$
ac	Acetate	$C_2H_3O_2{}^{-}$
pyr	Pyruvate	$C_3H_3O_3{}^{-}$
glc__D	D-Glucose	$C_6H_{12}O_6$
pro__L	L-Proline	$C_5H_9NO_2$

bon source individually. Potential carbon sources were defined as carbon-containing metabolites associated with an exchange reaction in the model (410 metabolites in total), and simulations were conducted across all strains included in the study. For each substrate, we calculated the fraction of strains predicted to grow. From this set, we selected 22 carbon sources listed in 3 spanning a broad range of growth prevalence across strains (Fig. 5C in Main Text, black bars), while maintaining a balanced representation of major compound classes, including carboxylic acids, amino acids, and carbohydrates.

1.1.4 Gapfilling metabolic models for coculture experiments'predictions

In coculture experiments performed by (Oña *et al.* [2]), the authors explored niche expansion for different strains comparing their growth in monoculture and in coculture. They considered both wild type strains, and auxotrophic strains for four amino-acids (leu, arg, trp and his). They grew these strains in MMAB having one of the 32 carbon sources available in biolog ecoplates, with

and without supplementation in the media of the KO amino-acid.

Influence of experimental criteria determining strains' growth

For each culture, they provided measurements of optical density (OD) at different days (samples taken after 0, 3, 5 and 7 days of growth) in triplicate. Importantly, they considered growth if at least one out of three biological replicates had an OD above 0.08. As we will show below, the accuracy of `misosoup` necessarily depends on these choices, so we systematically explored its accuracy as a function of variations in these parameters.

To compare the results with the experiment, we built for each combination of experimental criteria parameters c (OD threshold, number of replicates, and time), two binary vectors: v^i_{exp} and v^i_{FBA} where i corresponds to each of the acgam-gapfilled models of the three strains, describing growth ($= 1$) or not ($= 0$). By comparing these vectors, we estimate the extent to which the models perform like the experiments, what will affect downstream analysis.

The first test we conducted was to verify that, whenever growth was observed in monoculture experiments, the acgam-gapfilled models were able to reproduce it. Oña *et al.* considered as a criterion to determine growth, that an OD of 0.08 should be observed in at least one replicate. Therefore, we verified whether variations in this criteria influenced predictive performance. We considered different combinations c of OD thresholds, number of replicates and growth times to determine performance in acgam-gapfilled models. We found that, at day 0, the models showed high sensitivity for low thresholds when growth in 1 or 2 wells was considered, whereas sensitivity is low when 3 wells was considered, also having low specificity (Fig.1). This behaviour is expected, because bacteria had no time to grow. Hence, when compared with models' predictions, it translates into a high number of false positives and a low number of false negatives, hence lowering specificity but increasing sensitivity. In the remaining scenarios, the number of wells is the only factor that increases sensitivity, with no systematic impact on specificity.

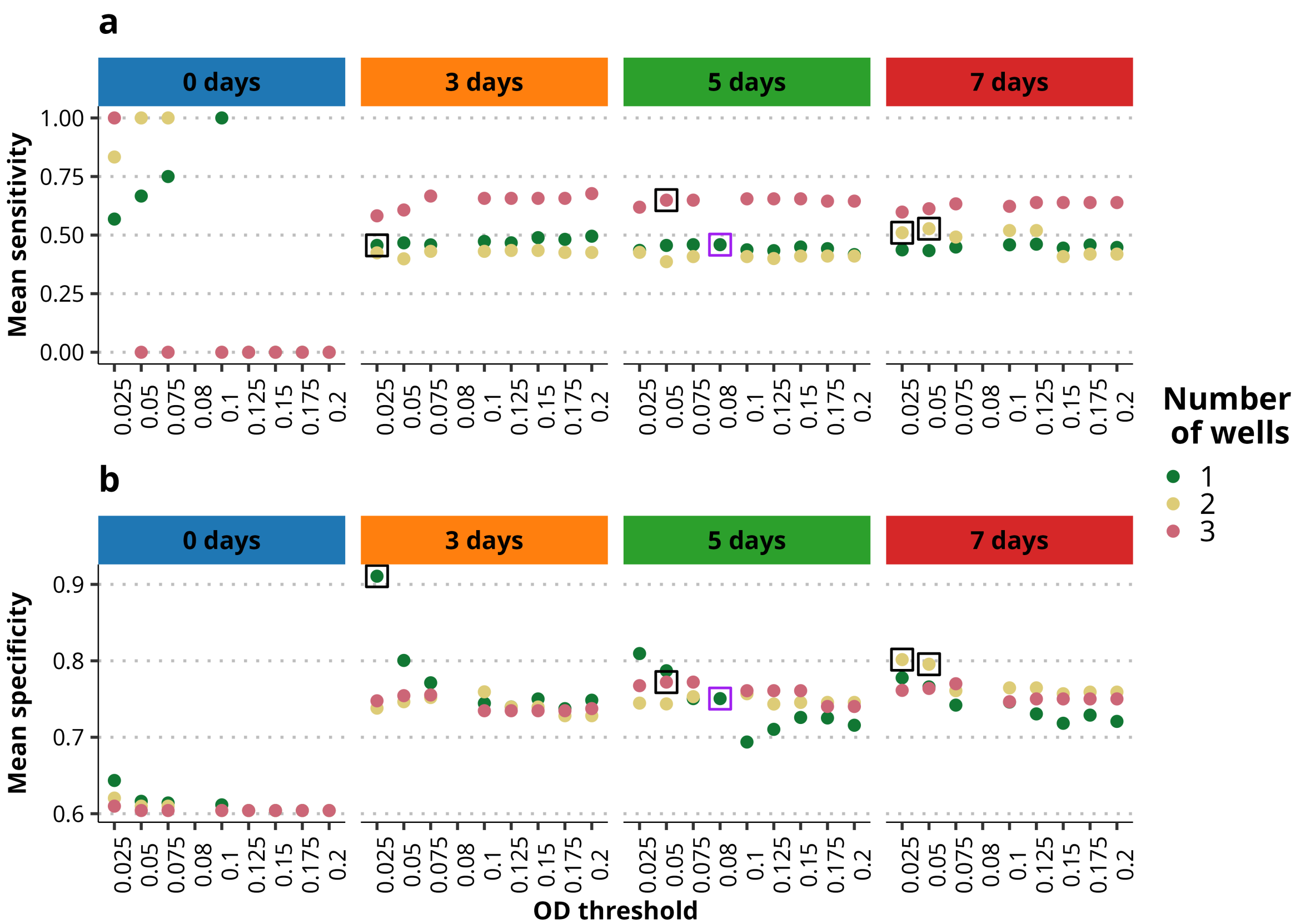

Figure 1: **Mean performance of metabolic models for different combinations of experimental parameters.** Performance was tested using acgam-gapfilled models. Selected combinations are indicated with black squares, with a purple one for Oña's *et al.* choice. The number of wells in which growth was observed is the factor that has the greatest influence on sensitivity, whilst the impact on specificity depends to a greater extent on the optical density from the third day onwards. The remaining factors do not affect the performance with the exception of time at day 0, where cultures have not achieved high ODs.

Influence of gapfilling in WT metabolic models performance

Since criteria to determine whether a species grows determines the media in which models should be gapfilled, we explored the effect of gapfilling depending on the selected combinations of parameters. We selected five c combinations, indicated with squares in Fig. 1. We chose one combination in which there was growth in all three wells with an intermediate OD, as replicability was the factor that most notably increases sensitivity. Then, we selected three scenarios with high specificity, because we expect gapfilling to preferentially increase sensitivity. Finally, we selected the scenario chosen by Oña *et al.* (purple square).

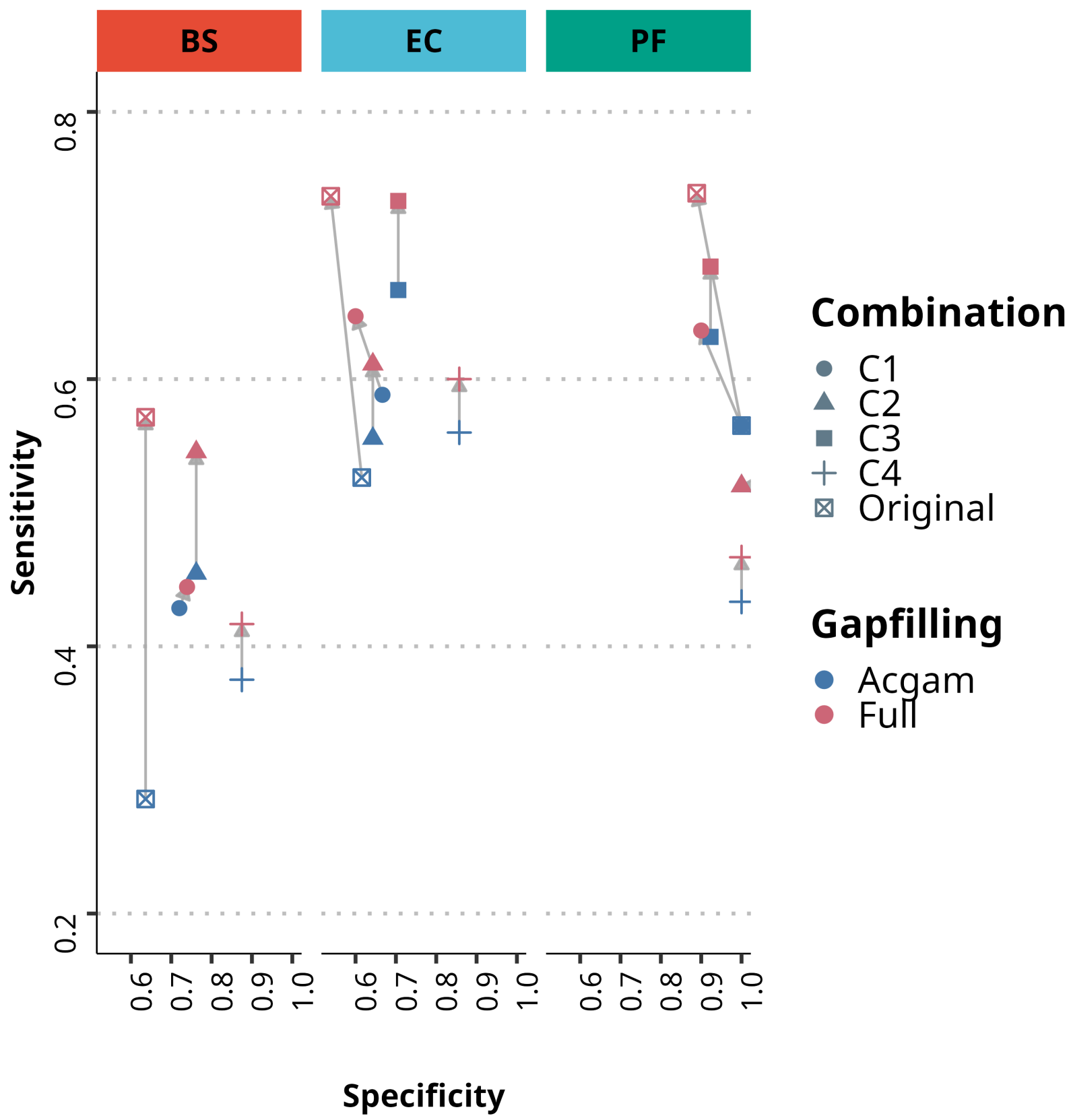

Figure 2: **Performance of selected experimental parameter combinations before and after gapfilling WT models**. Sensitivity and specificity for each acgam-gapfilled model before and after gapfilling show that gapfilling improved sensitivity, with little effect on specificity. BS had a notably lower initial sensitivity, suggesting the quality of the model was poorer. Despite improvement after gapfilling, it very likely affected its performance in coculture (also see Suppl. Fig. 4).

We gapfilled WT models considering the 5 combinations of experimental parameters selected. For this, we considered acgam-gapfilled WT models to explore which carbon sources considered in the experiment `misosoup` predicted growth when growth was indeed observed. Growth was determined using FBA with `cobrapy`, using the method `.optimize()`, and considering that the objective function value should be above 0.01. For each carbon source in which there was growth in the experimental data but not in our model, we further gapfilled the model for that specific C source using the gapfilling utility available in `carveme`. However, for 8 out of 32 C sources gapfilling led to thermodynamically infeasible cycles in which models were able to generate ATP without any source of energy in the medium. These C sources were itacon, ocdca, 4hbz, peamn, galctn D, glycogen, and eryth, so no gapfilling was considered in these media

As a final note, some carbon sources had no direct ID in BIGG, so we selected similar metabolites whenever possible (for example, for tween 40 or tween 80 the fatty acids without the ester were used as a proxy). In addition, ciclodextrin was not included since there was no reliable proxy in BiGG. This led to experiments on 31 carbon sources. After gapfilling, models' sensitivity improved with no effect on specificity, as expected (see Fig.2). The combination selected by the authors (indicated as 'original') was the one showing a higher increase in sensitivity. Note that, in C3, there was no growth for BS. Therefore, since there was no strong evidence of improvement to choose another scenario, for consistency with Oña's results we continued our analysis using the original combination of parameters.

Influence of `misosoup`'s objective function values in its accuracy

Finally, since `misosoup` predictions may also depend on the specific community growth threshold, g_c, we analysed whether it influences `misosoup` accuracy. For this analysis, we considered the 'original' combination of experimental parameters, although other choices led to qualitatively similar results (see below).

As was the case for individual models, to compare the results with the experiment we built, for each combination c of parameters determining whether growth was observed, two binary vectors v^{exp} and v^{mis}, describing observation ($= 1$) or absence ($= 0$) of growth in the experiment and in `misosoup` predictions, respectively.

We ran `misosoup` with the MSC algorithm without any community growth threshold in its version 2.3.0. We simulated all-against-all combinations of the four auxotrophs (H, R, L, W) for the 3 strains in 31 substrates, leading to 2046 simulations. To focus on `misosoup` performance and neglect errors that can objectively be attributed to the use of automatically-curated models, `misosoup` accuracy was computed on cases in which there was an agreement between experiments and prediction for auxotrophic strains grown in monoculture, when the amino-acid missing was supplemented in the medium.

Increasing g_c led to an almost constant trend in sensitivity and specificity until a threshold around 0.6 where there was a sharp drop in sensitivity and a less pronounced increase in specificity. Since, in light of the accuracy, the increase in specificity does not compensate the decrease in sensitivity, we conservatively chose a threshold of 0.5 for downstream analysis.

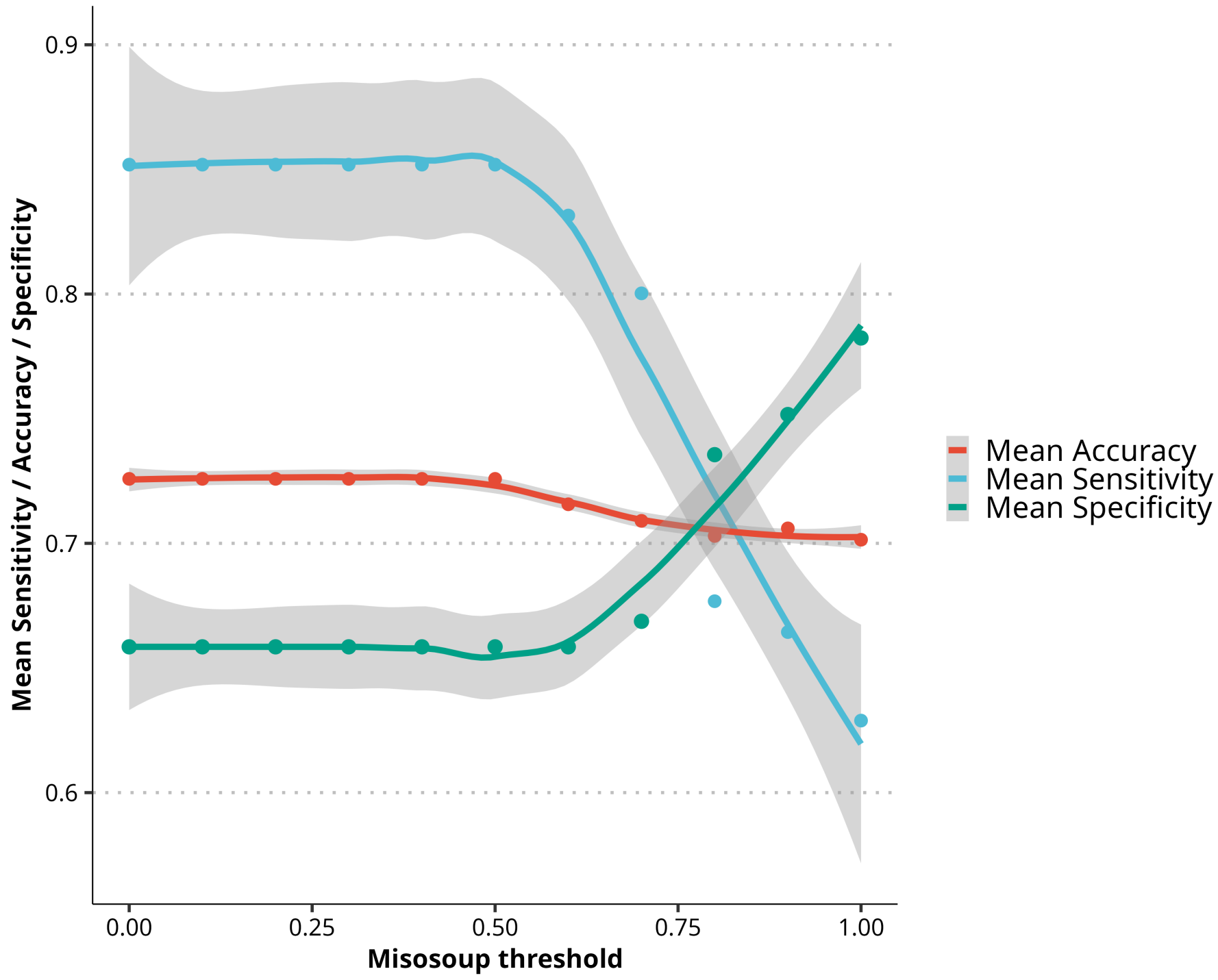

Figure 3: **Performance of `misosoup` for different community growth thresholds.** Although the mean accuracy is almost constant across the values of g_c; however, there is a clear trade-off between sensitivity and specificity for values of g_c above 0.75 with the accuracy remaining constant along the different community growth thresholds.

Consistency of gapfilling with `Carveme`

We tested whether gapfilling in `Carveme` depends on the specific run when several substrates were considered for gapfilling. After running 10 iterations for each model, we found that the reactions added were not identical in each iteration, although the ability of the final model to grow on the selected substrates remained the same. There was a set of core reactions added in all iterations and a set of variable reactions Fig.4. EC and PF were more consistent across iterations than BS was. Moreover, in one of the iterations (excluded in Fig. 4), gapfilling of BS introduces an infeasible cycle due to the addition of the H20tex transporter, which allowed the model to remove protons from the cell at no cost. These results pointed once again towards a lower quality of this model, which likely explains the higher number of false positives found in coculture experiments when it was included in the prediction.

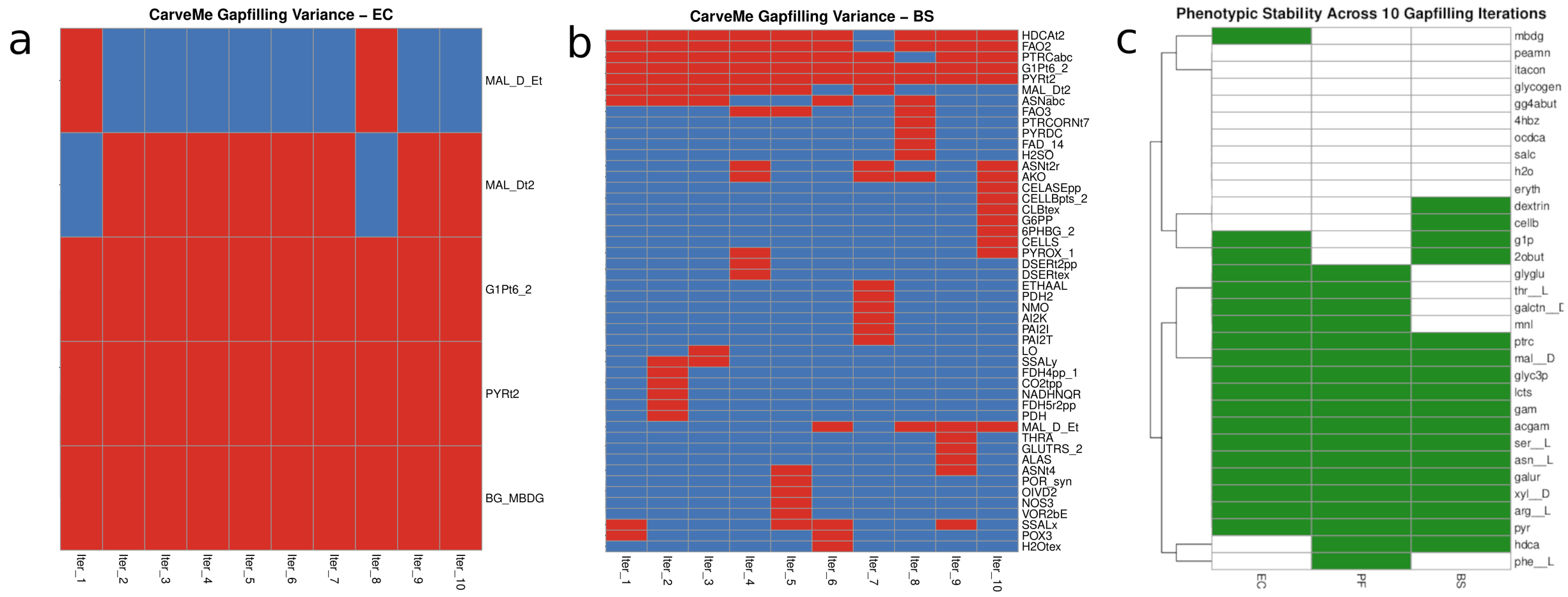

Figure 4: **Gapfilling consistency**. `Carveme` does not consistently add the same reactions when different runs are performed to gapfill the same media. Reactions added (red cells) in each of the 10 iterations performed for *E. Coli* (A) and *B. Subtilis* (B). BC model required gapfilling in more media, indicating that the model initially had a lower quality. Despite this variability, phenotypic plasticity was consistent (C), with models consistently growing in the same media in all 10 iterations (green cells). EC = *E. Coli*; PF = *P. fluorescens*; BS = *B. Subtilis*.

1.1.5 Working with minimal communities outside `misosoup`

The minimal communities identified by `misosoup`can be further analyzed using additional software packages. This is particularly useful when aiming to explore consumption and secretion profiles of each strain in the community under objectives beyond those implemented in `misosoup`(e.g., maximizing community biomass or minimizing total flux). Here, we provide an example of constructing a community model for a minimal community comprising three species and exporting it as an SBML file for compatibility with other tools.

```python
# Example script to recreate the community models with reframed and misosoup

from misosoup.reframed.layered_community import LayeredCommunity
from reframed.io.sbml import load_cbmodel, save_cbmodel

# Build community model for minimal community identified with misosoup
model_paths = [
    "A1R12.xml",
    "I2R16.xml"
]

```

```python
# Load the species models with reframed
models = [load_cbmodel(path, flavor="fbc2") for path in model_paths]

# Create the community model with misosoup's `LayerdCommunity` class.
# `LayerdCommunity` is a subclass of `Community` from reframed. It adds an
# additional layer of exchange reactions for the individual community members.
community = LayeredCommunity(community_id="community", models=models)

# Save the community as an SBML file with reframed. Note that the
# `reframed.CBModel` will be created lazily when reading the `merged_model`
# attribute.
save_cbmodel(community.merged_model, "community.xml")

```

1.2 Supplementary Results

1.2.1 Supplementary Note. Comparison of packages SMETANA and misosoup

To illustrate the difference between the analysis performed by SMETANA [3] and misosoup, we simulated two examples for each method. Results can be found in https://github.com/sirno/niche_expansion_data under the path: data/examples/comparison_smetana_misosoup.

For the first example we used strains Arcobacteraceae_A1R12, Alteromonadaceae_I2R16 and Vibrionaceae_I3M07 from the marine strains' dataset. First, we used misosoupto search for minimal supplying communities that would allow growth of Arcobacteraceae_A1R12 (the focal strain) in marine medium (see Method's section 1.1.3) with acetate as the sole source of carbon. We found two MSCs, with Alteromonadaceae_I2R16 or Vibrionaceae_I3M07 as supplying strains. misosoup's output returns the minimal supplying communities found, as well as a detailed description of each species consumption/secretion pattern and growth rate, compatible with maximal community growth.

Then, we used the same three species and medium composition and used SMETANA, in both global and detailed modes (see example1/smetana/run.sh). The global analysis returns two scores: metabolic interaction potential (mip) and metabolic resource overlap (mro). These quantify the number of nutritional requirements that the community can provide through cross-

feeding interactions, and the overlap in the minimal nutritional requirements, respectively. The detailed analysis returns three scores (scs, mus and mps) which are combined into a fourth score named SMETANA that quantifies the cross-feeding plasticity between the community members.

Note that for this first example, we could have inferred the two minimal supplying communities obtained with `misosoup`from the detailed analysis of SMETANA. In `example1/smetana/output_example1_detailed.tsv` we find that both Alteromonadaceae_I2R16 and Vibrionaceae_I3M07 can act as donors of metabolites which are required for growth of Arcobacteraceae_A1R12. However, this can only be easily deduced when no more than two strains participate in the interaction.

In the second example (see `example2/`) we used a set of three other strains (Vibrionaceae_1A01, Psychromonadaceae_B3M02 and Alteromonadaceae_D2M02) and simulated a marine medium (see Method's section 1.1.3) with D-glutamate (`glu__D`) as carbon source. First, we used `misosoup`to find a minimal supplying community that would allow growth of Psychromonadaceae_B3M02. We found that only when both Vibrionaceae_1A01 and Alteromonadaceae_D2M02 are present, Psychromonadaceae_B3M02 grows. In contrast, when we used SMETANA for the analysis of this community, both, the global and detailed analysis will not contain any results, because on this medium, none of the three strains can grow in isolation.

1.2.2 Supplementary Note. Additional results for coculture experiments

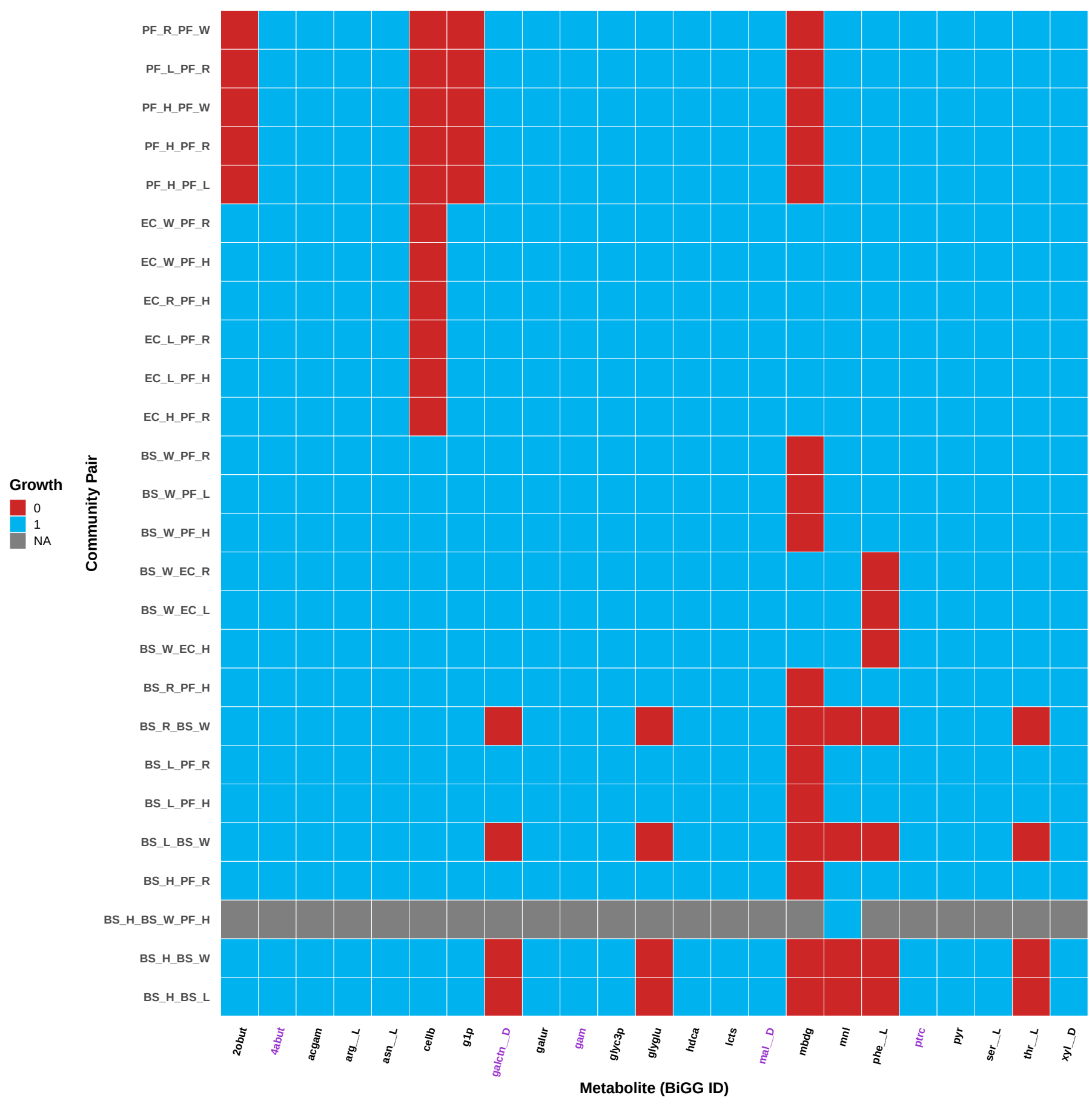

Figure 5: Additional coculture predictions obtained with `misosoup` that were not experimentally verified. Those involving BS should be taken with caution, given the false positives detected in previous analysis. Interestingly, `misosoup` also detects coculture pairs formed by pairs of auxotrophs of the same species.

1.2.3 Supplementary Note. Additional results for marine strains simulations

MSCs show high metabolic and niche complementarity with phylogenetic relatedness at or above the Family level

As we noted in Main Text, we found that the lower the phylogenetic distance, the less similar the reactions are between pairs of species (Suppl. Fig. 6, left), which translates into an also lower niche similarity (Suppl. Fig. 6, right). In addition, we also observed that, if the fundamental niche size is larger than zero, the higher the number of genes or reactions, the higher the fundamental niche size (Suppl. Fig. 7).

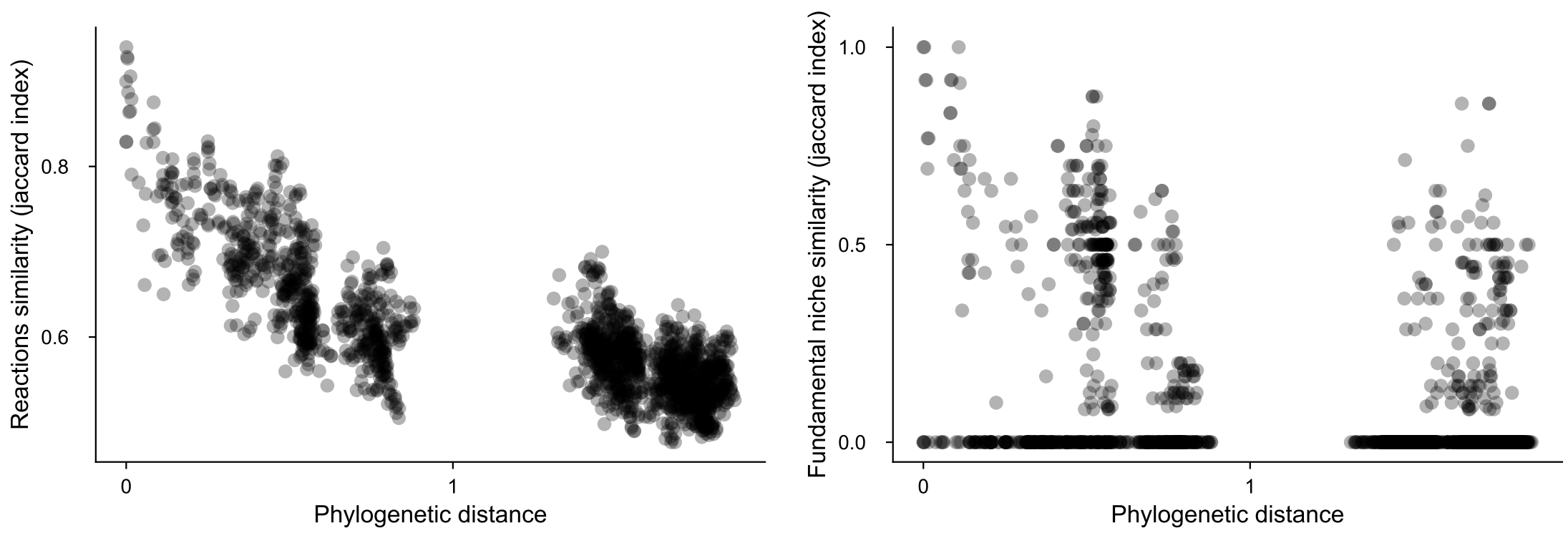

Figure 6: Relationship between phylogenetic distance and reactions similarity (left) and fundamental niche similarity (right). Each point represents a pair of strains. Reaction similarity is calculated as the Jaccard index, defined as the ratio of shared reactions to the total number of unique reactions (union of reactions).

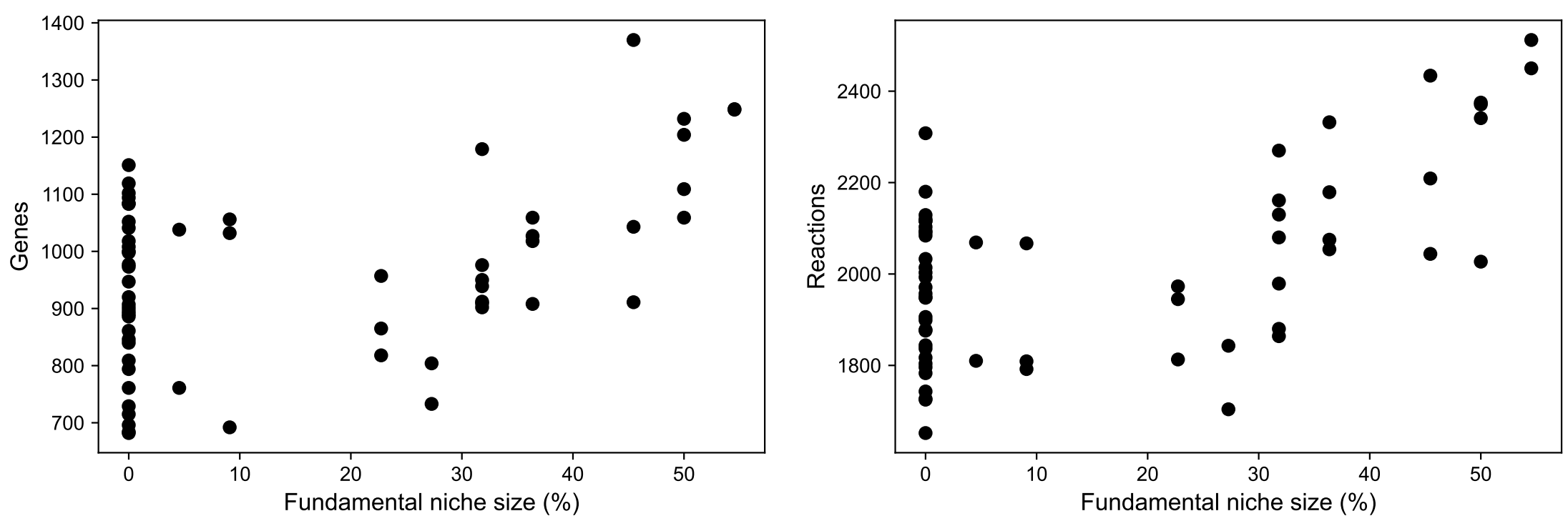

Figure 7: Species' fundamental niche vs number of genes (left) and reactions (right) in its metabolic model. Niche size is quantified as percentages of environments that a species thrives in.

Digging further into these observations, we investigated the phylogenetic relationships among members of minimal supplying communities (MSCs), initially focusing on 2-strain communities. We found that the strains forming MSCs predicted by `misosoup` are, on average, phylogenetically closer to each other than randomly selected strain pairs (Fig. 8A). Importantly, while MSCs showed increased phylogenetic relatedness compared to random communities, they were not exclusively composed of members from the same family – communities composed of strains from different families are also frequently observed. This general trend of phylogenetic relatedness held true for 3-strain MSCs as well, which were similarly composed of phylogenetically closer strains than randomly formed groups (Suppl. Fig. 10).

In [2], the authors investigated whether the overlap in strains' fundamental niches could

be used to predict the degree of niche expansion. Specifically, for every pair of strains they quantified the union minus the intersection (hereafter referred to as the symmetric difference) of the strains' fundamental niches. They found that, indeed, the symmetric difference between strains' fundamental niches could predict the degree of niche expansion, where a larger symmetric difference is indicative of a larger niche expansion.

Building on this observation, we calculated the symmetric difference of the fundamental niches of strains that constitute the predicted minimal supplying communities, and then compared these to the symmetric differences found in randomly selected sets of strains from the same species pool. We hypothesized that strains forming MSCs identified by `misosoup` would exhibit a larger symmetric difference in their fundamental niches.

Our findings confirmed this hypothesis. Consistent with results in [2], we observed that the symmetric difference of fundamental niches for the predicted communities was higher than that of random communities (Fig. 8B). Interestingly, this distinction between MSCs and random communities was primarily driven by a larger union in the fundamental niches (Suppl. Fig. 11) rather than by a smaller intersection.

Based on this observation we decided to investigate whether the differences between random and predicted communities appeared at genetic levels. For each minimal community, we considered the set of genes (or reactions) of each strain G_i and computed the union, intersection and symmetric differences of pairs or triplets of strains. For instance, the symmetric difference between two strains i and j is defined as the union minus the intersection $G_i \Delta G_j = (G_i \cup G_j)/(G_i \cap G_j)$. We tested the values found by building a random distribution by selecting pairs of strains randomly with replacement. Again, we observed that there was a significant difference between predicted and random communities, with random communities showing a lower symmetric difference in the metabolic reactions 8C.

Overall, we observed that MSCs were made up of pairs of strains from the same or different family with complementary sets of reactions. This complementarity in metabolic reactions is likely to explain the complementary fundamental niches of the strains involved. Importantly, this complementarity of metabolic reactions and fundamental niches is not observed in communities created at random from the same pool of strains.

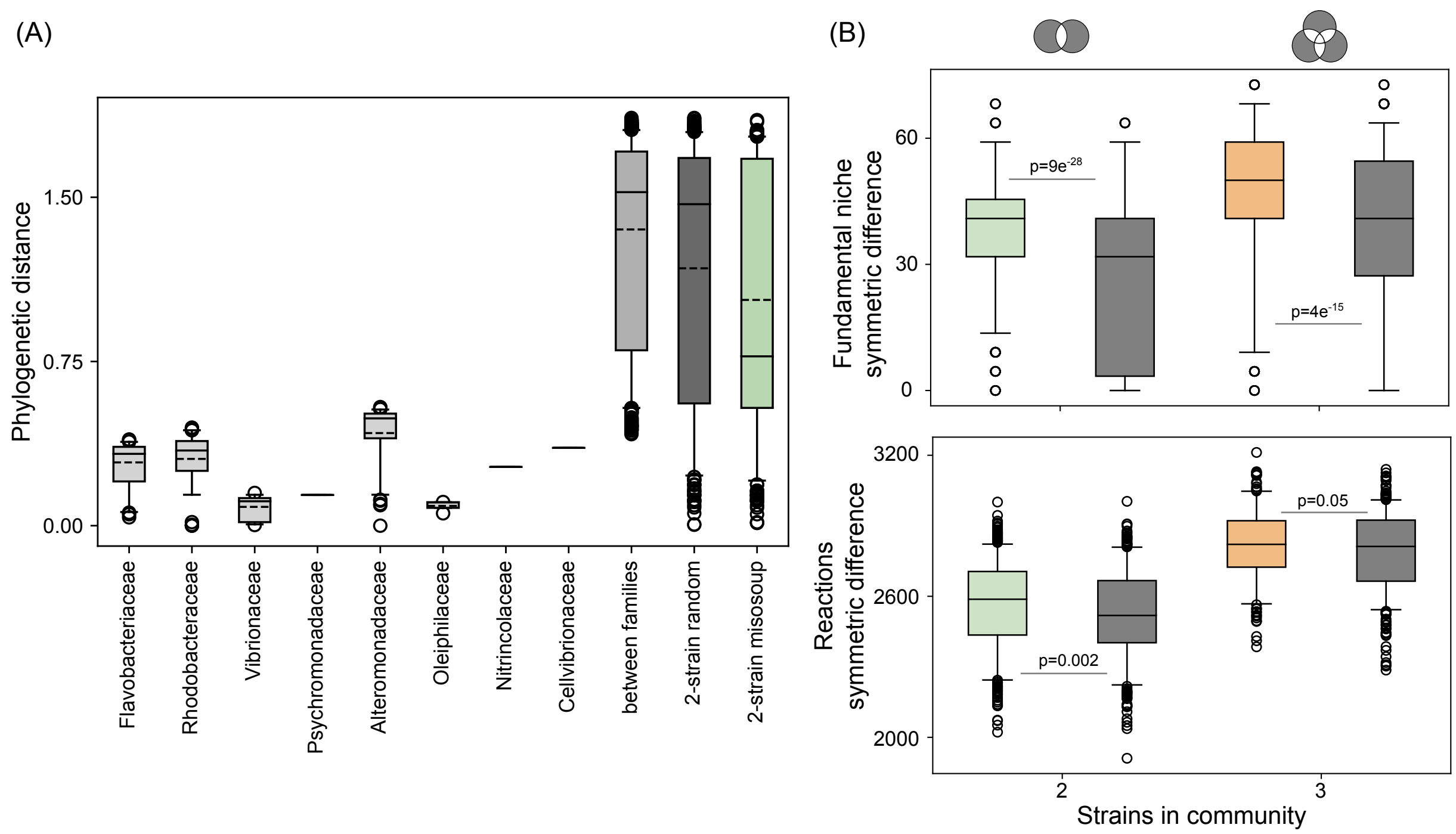

Figure 8: (A) Phylogenetic distances between pairs of strains within the same family (family names indicated on the x-axis), between pairs of strains from different families, between 500 randomly selected strain pairs, and between 2-strain communities predicted by `misosoup`. Symmetric difference of fundamental niches (B) and reactions in the metabolic models (C) for MSCs and 500 communities of random composition. Results for MSCs with two or three strains are shown in green and orange respectively. Results for random communities are shown in gray. Boxes extend from the first quartile to the third quartile of the data, with a line at the median.

Supplementary figures from marine strains simulations

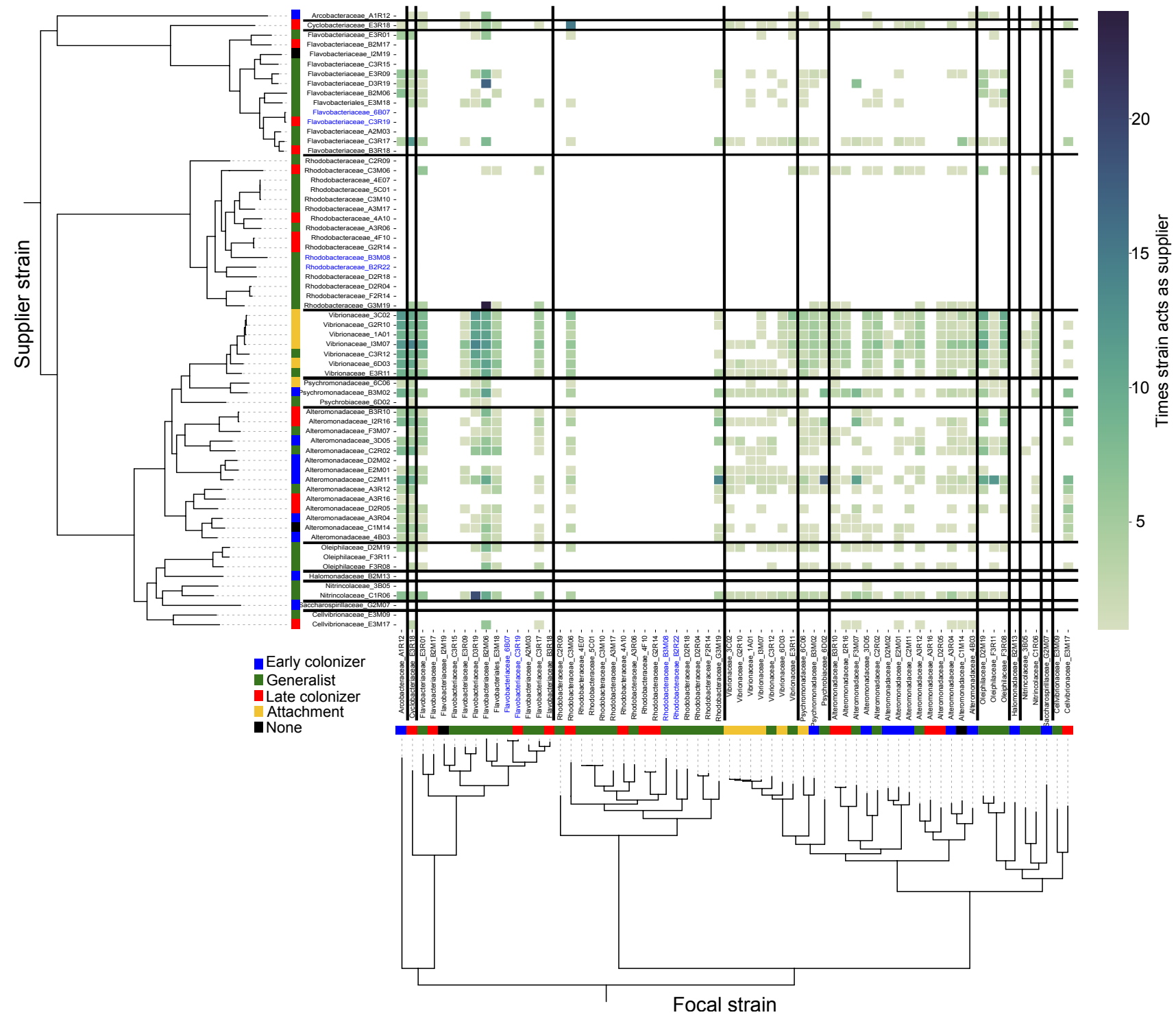

Figure 9: Heatmap depicting the times a strain acts as a supplier (y-axis) to a focal strain (x-axis). The phylogenetic tree and ecological strategies of the strains, as inferred in Pascual-Garcia (2022), are shown for both focal and supplier strains. Black lines indicate boundaries between strains from different families.

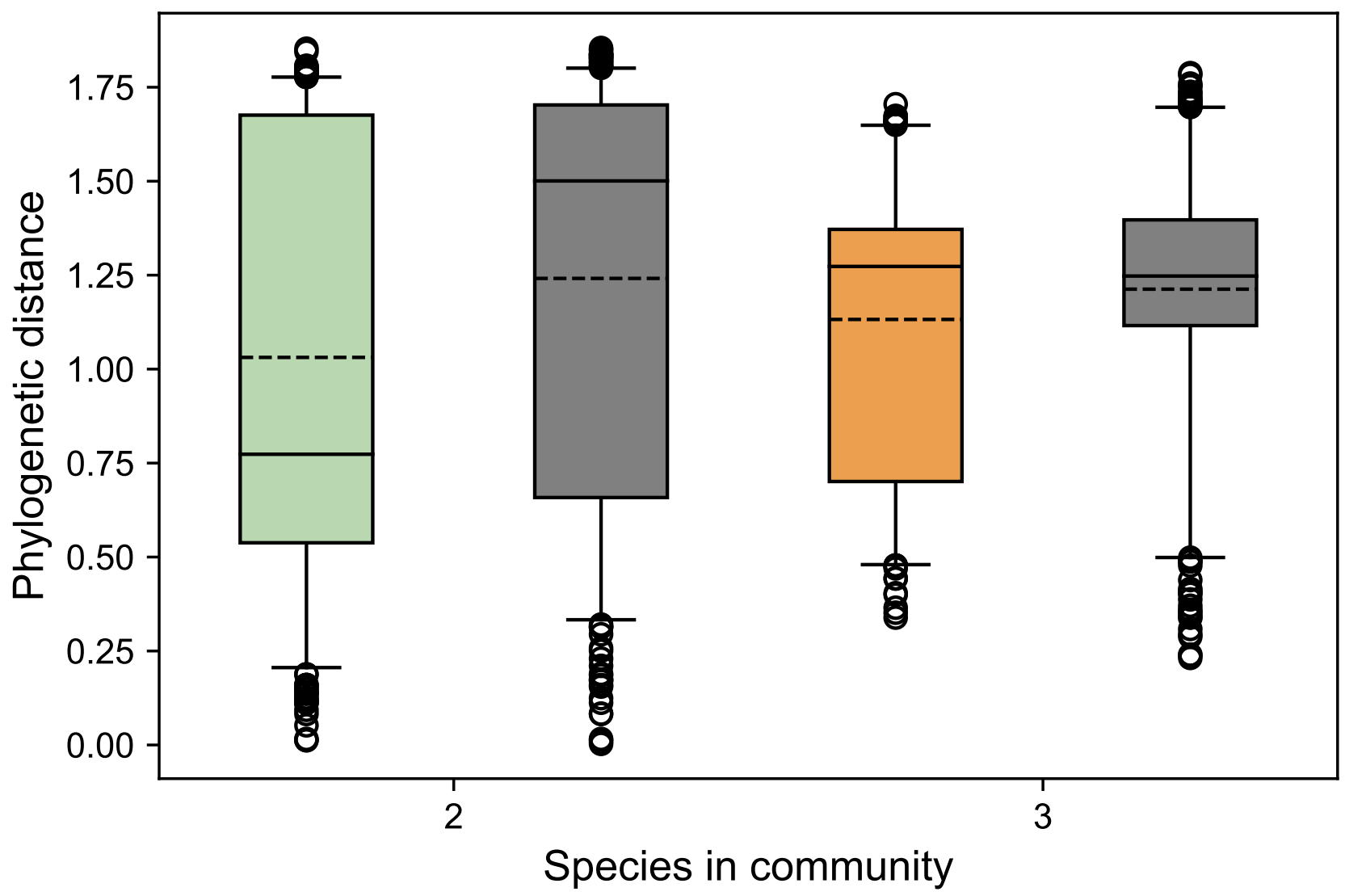

Figure 10: Phylogenetic distance for MSCs and 500 communities of random composition. Results for minimal supplying communities with two or three species are shown in green and orange respectively. Results for random communities are shown in gray. For communities made up of three species, the phylogenetic distance shows the average distance among the three possible pairs of species. Boxes extend from the first quartile to the third quartile of the data, with a continuous line at the median and a dashed line at the mean. Phylogenetic distance in MSCs is significantly smaller than that of communities built at random from the same species pool.

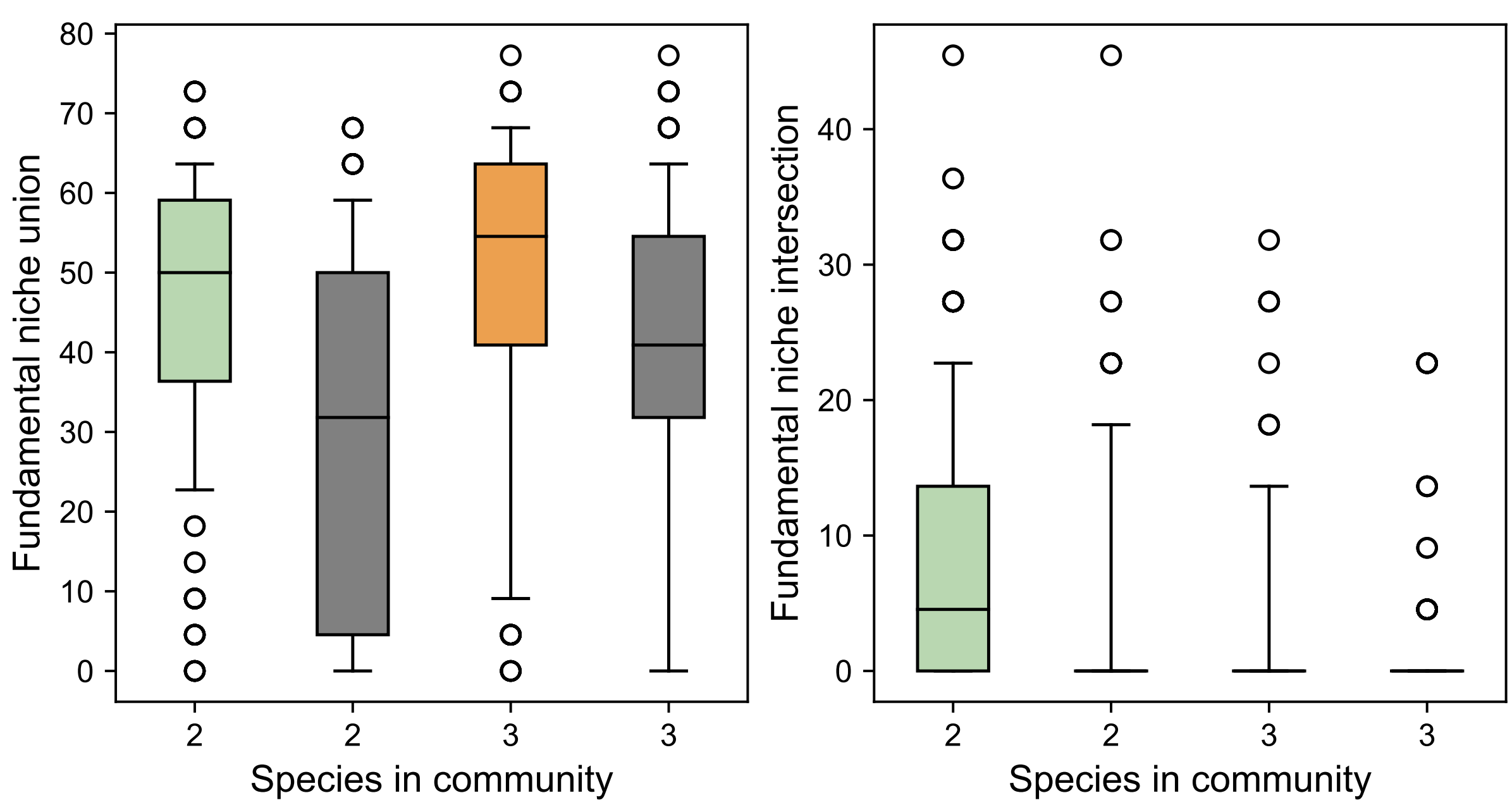

Figure 11: Fundamental niche union (left) and intersection (right) for minimal supplying communities and 500 communities of random composition. Results for MSCs with two or three species are shown in green and orange respectively. Results for random communities are shown in gray. Boxes extend from the first quartile to the third quartile of the data, with a line at the median.

References

[1] Orth, J. D. *et al.* A comprehensive genome-scale reconstruction of *Escherichia coli* metabolism–2011. *Molecular Systems Biology* **7**, 535 (2011).

[2] Oña, L. *et al.* Obligate cross-feeding expands the metabolic niche of bacteria. *Nature Ecology & Evolution* **5**, 1224–1232 (2021). Publisher: Nature Publishing Group.

[3] Zelezniak, A. *et al.* Metabolic dependencies drive species co-occurrence in diverse microbial communities. *Proceedings of the National Academy of Sciences* **112**, 6449–6454 (2015). Publisher: Proceedings of the National Academy of Sciences.
