# Supplementary material for: misosoup: a metabolic modeling tool for identifying minimal microbial communities, facilitates the exploration of microbial ecology and biotechnological applications
Source: mSystems. 2026 Jul 28;11(8):e00688-26. doi: 10.1128/msystems.00688-26 (PMC13483462; doi:10.1128/msystems.00688-26)
Supplement: Supplementary file 2 [file reviewer-comments.pdf]

AMERICAN SOCIETY FOR MICROBIOLOGY | mSystems®

misosoup: A metabolic modeling tool for identifying minimal microbial communities facilitates the exploration of microbial ecology and biotechnological applications.

Nicolas Ochsner, Magdalena San Román, Adrián Jiménez-Fernández, Sebastian Bonhoeffer, and Alberto Pascual-Garcia

Corresponding Author(s): Alberto Pascual-Garcia, Consejo Superior de Investigaciones Cientificas

Review Timeline:

Submission Date:	June 3, 2026
Editorial Decision:	June 11, 2026
Revision Received:	June 18, 2026
Accepted:	June 22, 2026

Editor: Babak Momeni

Reviewer(s): Disclosure of reviewer identity is with reference to reviewer comments included in decision letter(s). The following individuals involved in review of your submission have agreed to reveal their identity: Pranas Grigaitis (Reviewer #2)

Transaction Report:

(Note: With the exception of the correction of typographical or spelling errors that could be a source of ambiguity, letters and reports are not edited. The original formatting of letters and referee reports may not be reflected in this compilation.)

DOI: https://doi.org/10.1128/msystems.00688-26

Re: mSystems00688-26 (misosoup: A metabolic modeling tool for identifying minimal microbial communities provides valuable insights into microbial ecology and biotechnological applications.)

Dear Dr. Alberto Pascual-Garcia:

Thank you for the privilege of reviewing your work. Below you will find my comments, instructions from the mSystems editorial office, and the reviewer comments.

The reviewers agree that the revisions in scope and narrative have improved the manuscript. There are, however, some important edits still needed before the manuscript is ready for publication. Please refer to the reviewers' comments below for their suggestions.

Please return the manuscript within 60 days; if you cannot complete the modification within this time period, please contact me. If you do not wish to modify the manuscript and prefer to submit it to another journal, notify me immediately so that the manuscript may be formally withdrawn from consideration by mSystems.

Revision Guidelines
To submit your modified manuscript, log into the submission site at https://msystems.msubmit.net/cgi-bin/main.plex. Go to Author Tasks and click the appropriate manuscript title to begin. The information you entered when you first submitted the paper will be displayed; update this as necessary. Note the following requirements:

• Upload point-by-point responses to the issues raised by the reviewers in a file named "Response to Reviewers," NOT in your cover letter.
• Upload a compare copy of the manuscript (without figures) as a "Marked-Up Manuscript" file.
• Upload a clean .DOC/.DOCX version of the revised manuscript and remove the previous version.
• Each figure must be uploaded as a separate, editable, high-resolution file (TIFF or EPS preferred), and any multipanel figures must be assembled into one file.
• Any supplemental material intended for posting by ASM should be uploaded with their legends separate from the main manuscript. You can combine all supplemental material into one file (preferred) or split it into a maximum of 10 files with all associated legends included.

For complete guidelines on revision requirements, see our Submission and Review Process webpage. Submission of a paper that does not conform to guidelines may delay acceptance of your manuscript.

Data availability: ASM policy requires that data be available to the public upon online posting of the article, so please verify all links to sequence records, if present, and make sure that each number retrieves the full record of the data. If a new accession number is not linked or a link is broken, provide mSystems production staff with the correct URL for the record. If the accession numbers for new data are not publicly accessible before the expected online posting of the article, publication may be delayed; please contact production staff (mSystems@asmusa.org) immediately with the expected release date.

Publication Fees: For information on publication fees and which article types are subject to charges, visit ourwebsite. If your manuscript is accepted for publication and any fees apply, you will be contacted separately about payment during the production process; please follow the instructions in that e-mail. Arrangements for payment must be made before your article is published.

ASM Membership: Corresponding authors may join or renew ASM membership to obtain discounts on publication fees. Need to upgrade your membership level? Please contact Customer Service at Service@asmusa.org.

The ASM Journals program strives for constant improvement in our submission and publication process. Please tell us how we can improve your experience by taking this quick Author Survey.

Thank you for submitting your paper to mSystems.

Sincerely,
Babak Momeni
Editor
mSystems

Reviewer #1 (Comments for the Author):

Overall, the manuscript has improved. Few additional points (essential but minor):

1. Title: 'valuable insights' is vague, subjective, and also inaccurate (as the bar in biology for valuable insights is quite high).
2. "knocked out a set of strains..to generate.. auxotrophs" -> you mean used gene KOs? Please revise thoroughly for biological accuracy
3. Cross-feeding does not necessarily need bi-directional interaction. Please ensure that all used terminology is consistent with the ecological norms.

Reviewer #2 (Comments for the Author):

I thank the Authors for extensively revising the manuscript, and I believe that changing the angle towards the application potential of the package, rather than addressing a vast biological problem of niche expansion, was a right call from the Authors' side. The new use cases like the biogas-producing community are in particular appreciated.

My only (minor) request is to thoroughly scan the text once again for minor issues/typos, e.g. C02 (C-zero-two) as CO_2 in the Figure 3 etc., but these imperfections should be fixed during the copyediting anyways.

Pranas Grigaitis

Manuscript: mSystems00688-26
Title: misosoup: A metabolic modeling tool for identifying minimal microbial communities provides valuable insights into microbial ecology and biotechnological applications.

Dear Dr. Momeni,

Thank you very much for handling our manuscript and for the opportunity to publish it in mSystems. We would also like to thank the reviewers for assessing the revised version several months after the first review was carried out. We have taken all the comments into account in the new version, which we list below.

Reply to Reviewer #1

Overall, the manuscript has improved. Few additional points (essential but minor):

AUTHORS: We thank the reviewer for taking the time to evaluate the new version and for the positive feedback.

1. Title: 'valuable insights' is vague, subjective, and also inaccurate (as the bar in biology for valuable insights is quite high).

AUTHORS: Thank you for pointing out this issue, for which I am responsible, as, although I can immediately see how vague the expression is, I was unaware that it was linked to matters of such significant impact. In the new version we substiututed “provides valuable insights” for “facilitates the exploration of”:

misosoup: A metabolic modeling tool for identifying minimal microbial communities facilitates the exploration of microbial ecology and biotechnological applications.

2. "knocked out a set of strains..to generate.. auxotrophs" -> you mean used gene KOs? Please revise thoroughly for biological accuracy.

AUTHORS. Apologies for the lack of precision. The new sentence is “We considered an experiment by Oña et al. [30], in which gene deletions were introduced to generate amino acid auxotrophs.”

3. Cross-feeding does not necessarily need bi-directional interaction. Please ensure that all used terminology is consistent with the ecological norms.

AUTHORS: We detected a wrong sentence that was corrected. In the remaining cases, we explicitly distinguish between unidirectional vs. bidirectional (or mutual) cross-feeding.

Reply to Reviewer #2

I thank the Authors for extensively revising the manuscript, and I believe that changing the angle towards the application potential of the package, rather than addressing a vast biological problem of niche expansion, was a right call from the Authors' side. The new use cases like the biogas-producing community are in particular appreciated.

My only (minor) request is to thoroughly scan the text once again for minor issues/typos, e.g. C02 (C-zero-two) as CO_2 in the Figure 3 etc., but these imperfections should be fixed during the copyediting anyways.

Pranas Grigaitis

AUTHORS: We than Dr. Grigaitis for the detailed review and for his final comments. We corrected the typo in Fig. 3 and reviewed the MS. We avoided the use of “species” when “strain” was more appropriate, and searched and corrected other minor inaccuracies, in addition to those highlighted by reviewer 1.

Re: mSystems00688-26R1 (misosoup: A metabolic modeling tool for identifying minimal microbial communities facilitates the exploration of microbial ecology and biotechnological applications.)

Dear Dr. Alberto Pascual-Garcia:

Your manuscript has been accepted, and I am forwarding it to the ASM production staff for publication. Your paper will first be checked to make sure all elements meet the technical requirements. ASM staff will contact you if anything needs to be revised before copyediting and production can begin. Otherwise, you will be notified when your proofs are ready to be viewed.

Data Availability: ASM policy requires that data be available to the public upon online posting of the article, so please verify all links to sequence records, if present, and make sure that each number retrieves the full record of the data. If a new accession number is not linked or a link is broken, provide production staff with the correct URL for the record. If the accession numbers for new data are not publicly accessible before the expected online posting of the article, publication may be delayed; please contact ASM production staff immediately with the expected release date.

Publication Fees: For information on publication fees and which article types have charges, please visit our website. We have partnered with Copyright Clearance Center (CCC) to collect author charges. If fees apply to your paper, you will receive a message from no-reply@copyright.com with further instructions. For questions related to paying charges through RightsLink, please contact CCC at ASM_Support@copyright.com or toll free at +1-877-622-5543. CCC makes every attempt to respond to all emails within 24 hours.

ASM Membership: Corresponding authors may join or renew ASM membership to obtain discounts on publication fees. Need to upgrade your membership level? Please contact Customer Service at Service@asmusa.org.

Embargo Policy: A press release may be issued as soon as the manuscript is posted on the mSystems Latest Articles webpage. The corresponding author will receive an email with the subject line "ASM Journals Author Services Notification" when the article is available online.

Cover Image Submissions: If you would like to submit a potential Cover Image, please email a file and a short legend to msystems@asmusa.org. Please note that we can only consider images that (i) the authors created or own and (ii) have not been previously published. By submitting, you agree that the image can be used under the same terms as the published article. Image File requirements: TIF/EPS, 7.5 inches wide by 8.25 inches tall (at least 2,250 pixels wide by 2,475 pixels tall), minimum 300 dpi resolution (600 dpi preferred), RGB, and no figure elements, e.g., arrows or panel labels. The legend should be a short description of the image, 1-2 sentences recommended. Please download and use this interactive template in Adobe to ensure that your proposed cover image meets our size requirements (https://journals.asm.org/pb-assets/pdf-text-excel-files/ASM-Interactive-Sizing-Cover-Template-1715689791.pdf).

Thank you for submitting your paper to mSystems.

Sincerely,
Babak Momeni
Editor
mSystems
